# A general and accessible approach to enrichment and characterisation of natural anti-Neu5Gc antibodies from human samples†

**DOI:** 10.1039/d5cb00073d

**Published:** 2025-05-15

**Authors:** Esme Hutton, Yumiko Uno, Emma Scott, Craig Robson, Martin A. Fascione, Nathalie Signoret

**Affiliations:** a Department of Chemistry, University of York York UK martin.fascione@york.ac.uk; b Hull York Medical School, University of York York UK nathalie.signoret@york.ac.uk; c Newcastle University, Centre for Cancer Newcastle UK

## Abstract

*N*-Glycolylneuraminic acid (Neu5Gc) is a non-human sialic acid which is presented on the surface of human cells following uptake from dietary sources. Antibodies against Neu5Gc have implications for many aspects of human health such as inflammation, xenograft rejection and cancer. However, current methods to detect and study anti-Neu5Gc antibodies require complex synthesis of glycan structures, animal handling expertise, or access to expensive reagents and equipment. Here, we outline a simple workflow to enrich and detect anti-Neu5Gc antibodies from small volume human serological samples. This strategy involves a micro-scale affinity purification step, followed by an indirect ELISA detection step which uses CMAH-transfected human cells as a source of Neu5Gc-containing human glycans in their native context. Parental wild type cells are also used as a paired Neu5Gc-negative control. Using this workflow, Neu5Gc-specific antibodies could be enriched from intravenous immunoglobulin (IVIG) and individual plasma specimens from ten healthy donors. Anti-Neu5Gc antibodies were detected in all donors, regardless of age or sex. The lysate ELISA assay was also sufficiently sensitive to observe reproducible individual differences in the anti-Neu5Gc reactivity of each donor specimen. Importantly, despite this individual variation, enriched antibodies from all donor specimens bound effectively to Neu5Gc-containing glycans presented on the surface of whole human cells, highlighting the potential physiological relevance of these antibodies.

## Abstract 2

A workflow to enrich and detect anti-Neu5Gc antibodies from individual plasma specimens, using CMAH-transfected human cells as a source of Neu5Gc-containing glycans.

## Graphical abstract

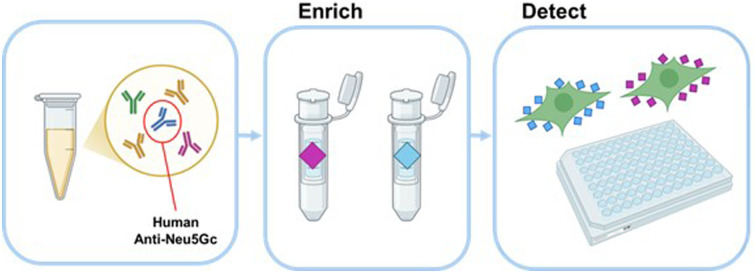

## Introduction

**Fig. 1:**
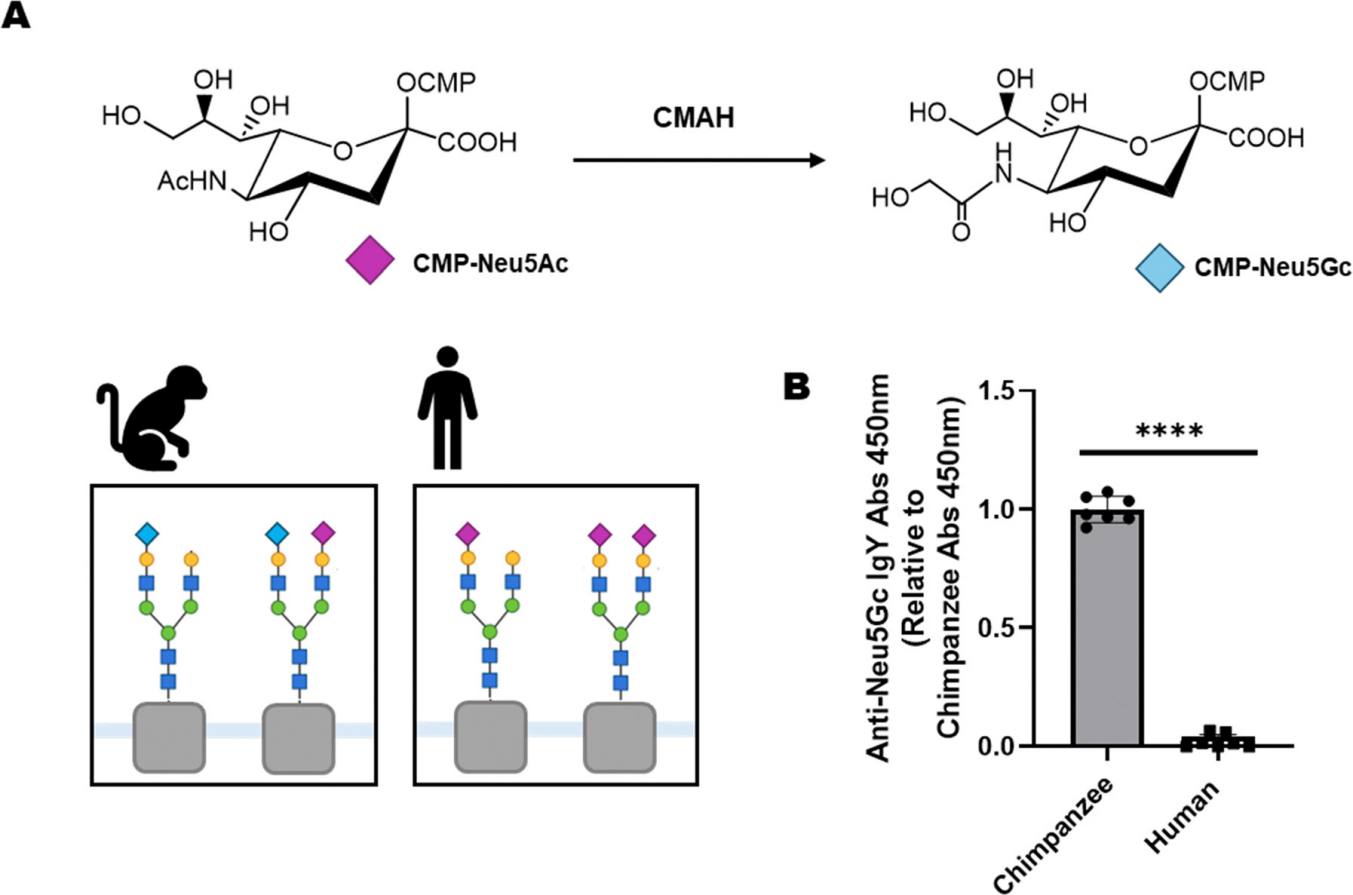
Neu5Gc is not endogenously synthesised by humans. (A) CMP-activated Neu5Ac is converted into CMP-Neu5Gc in an irreversible hydroxylation reaction catalysed by the enzyme CMAH. Chimpanzees have functional CMAH and endogenously synthesise both Neu5Ac (purple diamond) and Neu5Gc (blue diamond). Neu5Gc production is absent in humans due to CMAH inactivation. (B) Chimpanzee or human serum were adsorbed directly onto 96-well ELISA plates and polyclonal chicken anti-Neu5Gc IgY antibody binding to the sera was detected with a secondary HRP-conjugated anti-chicken IgY. The graph depicts mean ± SD anti-Neu5Gc IgY abs 450 nm, normalised to average abs 450 nm value for chimpanzee serum coated wells for 3 independent experiments (*P* = 0.0001, unpaired *t*-test).

Sialic acids are 9-carbon nonulosonic acid sugars predominantly found on the terminal end of cell surface glycans. This position at the outermost limits of the glycocalyx makes sialic acids key ligands for many glycan-binding proteins, such as viral lectins, bacterial toxins and inhibitory siglecs on immune cells.^1,2^ As such sialylation is implicated in many biological processes, in both health and disease.^3^ Mammals produce two main sialic acid species: *N*-acetylneuraminic acid (Neu5Ac) and *N*-glycolylneuraminic acid (Neu5Gc)^4^ (Fig. 1A). However, conserved loss-of-function mutations in the enzyme cytidine-monophospho-*N*-acetylneuraminic acid hydroylase (CMAH), which is essential for Neu5Gc synthesis, have led to an absence of endogenous Neu5Gc production in humans.^5^

Despite no evidence for an alternative biosynthetic pathway,^6^ Neu5Gc can still be detected at low levels in some human tissues, particularly those with high metabolic activity and sialic acid turnover such as the epithelia, foetal tissues, and cancer cells.^7^ This is due to uptake and incorporation of Neu5Gc from exogenous sources, such as red meat or dairy in the diet.^8,9^ Presentation of Neu5Gc on human sialoglycans is hypothesised to trigger the production of anti-Neu5Gc antibodies.^10,11^ These Neu5Gc-directed antibodies arise in early life,^12^ and are proposed as a driving factor in many inflammation-associated conditions such as atherosclerosis, xenograft rejection and cancer.^13–15^

Despite wide exploration of their role in human health, there is a lack of agreement surrounding the extent to which anti-Neu5Gc antibodies are present in the human population.^16^ This variation in the reported prevalence of anti-Neu5Gc antibodies may be linked to the absence of a general method for their detection and study. Enzyme-linked immunosorbent assays (ELISAs) detecting antibody binding to homogenous synthetic glycans have high sensitivity, but presenting a single glycan structure is not representative of the complex mixtures of linkage types and branches found in natural glycans.^17^ Glycan microarrays may overcome this problem by displaying Neu5Gc conjugated to a range of underlying glycan structures, but these platforms often do not accommodate the multivalent presentation of glycans in biological contexts.^18^ Flow cytometry has also been used to investigate anti-Neu5Gc binding to cell surface glycans, mostly with the aim of understanding xenograft rejection. These studies frequently use porcine cells which express different glycan profiles to those found in humans.^19,20^ Another key issue with many of these methods is that they require specialist equipment or expertise, including chemical/chemoenzymatic synthesis of complex glycan structures, animal handling, or microarray fabrication and derivatisation apparatus. As a result, study of anti-Neu5Gc antibodies is often not accessible to biomedical research groups.

To address these issues, we have developed a simple strategy to enrich anti-Neu5Gc antibodies out of small-volume serological specimens, and an ELISA method, which can be used to characterise the ability of these antibodies to bind to human Neu5Gc-containing glycans. This strategy utilises commercially available reagents, and use of cell lysates rather than whole cells may also extend its accessibility even to laboratories without access to cell culture facilities. It is anticipated that this workflow will broaden the accessibility of research into the anti-Neu5Gc antibody response, furthering understanding of the complex role these antibodies play in human health.

## Results and discussion

### Background and method design

Chimpanzee serum (CS) glycoproteins are similar in structure and composition to human serum (HS) glycoproteins.^21^ Unlike humans, however, chimpanzees express functional CMAH and produce a wide range of Neu5Gc-containing glycans. This was shown experimentally by adsorbing CS and HS onto a 96-well ELISA plate and assessing overall Neu5Gc content using a commercial polyclonal chicken anti-Neu5Gc IgY antibody (Fig. 1B). Enrichment of anti-Neu5Gc antibodies from serological samples has been reported previously using an affinity purification process which exploits this, using HS and CS as a paired system of Neu5Gc-negative and Neu5Gc-positive matrices. The affinity column system has been used to enrich anti-Neu5Gc antibodies from IVIG and pooled human serum. These antibodies were tested on sialoglycan microarrays,^22,23^ not accounting for glycan presentation in a natural context. This approach also uses large sample volumes not compatible with routine serological specimens, and therefore requires adaptations for micro-scale purification.

A key advance in the detection of anti-Neu5Gc antibodies was the development of a CMAH knockout mouse model that does not endogenously synthesize Neu5Gc.^24^ This model was then used to develop an ELISA protocol where wild-type (WT) mouse serum was used as a source of Neu5Gc-containing glycans, and CMAH knock-out (CMAH KO) mouse serum was used as a paired Neu5Gc-negative control.^25^ While effective, this strategy requires access to animal handling expertise, and mouse glycan structures may not represent the full range of Neu5Gc-containing glycans possibly found in human tissues.^26^ A more physiologically relevant alternative is to re-introduce CMAH activity in human cells, an approach already shown to enable binding of human Neu5Gc-reactive IgGs to transfected cells in flow cytometry experiments.^27,28^ However, studies investigating neuraminidase cleavage of surface sialic acids in various cell lines reported that up to a third of total sialic acid content may be intracellular, for example as part of the sialoglycoconjugates undergoing processing in the golgi before presentation on the cell surface.^29–31^ Flow cytometry experiments only account for cell surface glycans, and therefore may not detect the full range of Neu5Gc-reactive antibodies produced by humans. This presents the need for a parallel strategy which accounts for total cellular Neu5Gc content.

Based on this knowledge, we proposed that paired Neu5Gc-positive lysates from CMAH-transfected HEK 293 cells (mCMAH-HEK) and Neu5Gc-negative lysates from parental cells (WT-HEK) could be used on an ELISA platform for detection of specific antibody binding to human Neu5Gc-containing glycans. This strategy uses physiologically relevant glycan structures, accounts for total cell sialic acid content, and can be applied to multiple samples in a high-throughput manner. We also aimed to explore whether the pair of WT-HEK and mCMAH-HEK lysates could represent a more cost-effective alternative to using HS and CS for the affinity column enrichment of human anti-Neu5Gc antibodies from serological specimens.

### mCMAH-HEK development

**Fig. 2:**
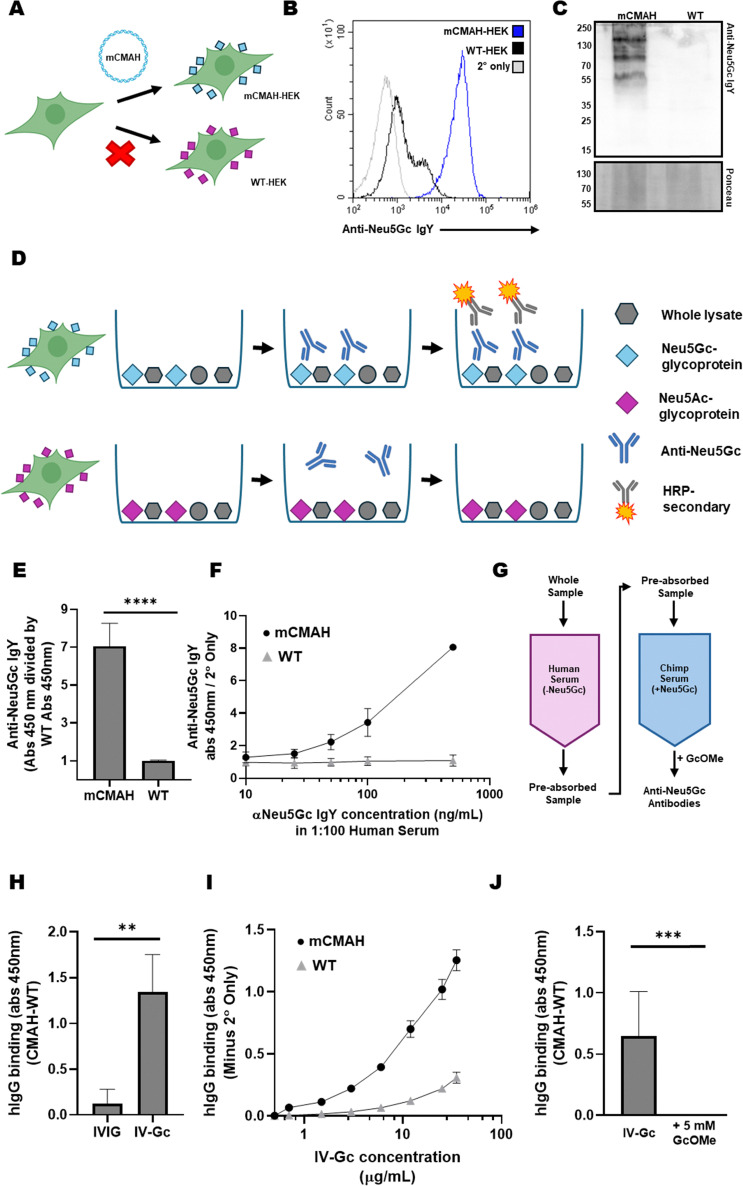
Development of a lysate ELISA and affinity purification strategy for anti-Neu5Gc antibodies. (A) HEK 293 cells (WT-HEK) do not express functional CMAH but can be transfected with mouse CMAH cDNA to convert Neu5Ac into Neu5Gc. (B) Neu5Gc production by mCMAH-HEK was confirmed by flow cytometry using a polyclonal chicken IgY anti-Neu5Gc antibody and an Alexa-488 conjugated anti-chicken IgY secondary. (C) Neu5Gc glycoconjugates were detected in mCMAH-HEK total cell lysate by westerm blotting using the same anti-Neu5Gc IgY antibody and an HRP-conjugated anti-chicken IgY secondary antibody. Ponceau staining (lower panel) was used to indicate total protein loading. 5 μg of lysate was loaded into each lane and images were taken at an exposure of 2.5 s. (D) A schematic depicting the lysate ELISA process. Whole lysates from mCMAH-HEK (upper) and WT-HEK (lower) are adsorbed onto 96-well ELISA plates. Anti-Neu5Gc IgY is added to the wells as the primary antibody and its binding is detected using an HRP-conjugated anti-IgY secondary antibody (figure produced in BioRender). (E) Anti-Neu5Gc IgY at 500 ng mL^−1^ was added to wells coated with mCMAH- or WT-HEK lysates and detected using an HRP-conjugated anti-chicken IgY secondary. Datapoints depict mean ± SD (*N* = 5, 2 independent experiments, unpaired *t*-test, *p* < 0.0001). (F) A titration of anti-Neu5Gc IgY spiked into 1 : 100 commercial human serum was added to mCMAH-HEK or WT-HEK lysate coated ELISA plates and detected using an anti-chicken IgY secondary. Datapoints depict mean ± SD (*N* = 5, 2 independent experiments). (G) A schematic outlining the CS/HS affinity purification method used to enrich anti-Neu5Gc antibodies from ImmunoVenin Intact IVIG. The whole sample is pre-cleared on the Neu5Gc-negative HS column, and anti-Neu5Gc antibodies are eluted from the Neu5Gc-positive CS column using a Neu5Gc α-methyl glycoside (GcOMe). (H) Whole IVIG and anti-Neu5Gc antibodies enriched from IVIG both at 10 μg mL^−1^ were tested on the lysate ELISA at 10 μg mL^−1^ and detected using an HRP-conjugated anti-human IgG secondary at 1 : 5000. Graph shows mean ± SD (*N* = 6, 2 independent experiments unpaired *t*-test, *P* = 0.0018). (I) IV-Gc concentration was determined using a BCA assay and added to the lysate ELISA at a titration from 0 to 35 μg mL^−1^. Human IgG binding was detected using an HRP-anti-human IgG antibody. Graph depicts mean ± SD (*N* = 3). (J) IV-Gc at 5 μg mL^−1^ was co-incubated with a competing dose (5 mM) of GcOMe then tested on the lysate ELISA. Binding was detected using an HRP-anti-human IgG antibody. For the GcOMe condition, the secondary only wells were also treated with 5 mM GcOMe. Graph shows mean ± SD (*N* = 5, 2 independent experiments, unpaired *t*-test, *p* = 0.001).

HEK 293 cells were chosen as a model to develop a paired system of Neu5Gc-positive CMAH-transfected cells. Neu5Gc-negative parental HEK cells were used as a control for background non-Neu5Gc antibody binding. HEK 293 cells were used due to their ability to express recombinant proteins,^32^ alongside published reports that HEK cells express many of the human glycosylation genes, leading to production of a diverse range of sialoglycan structures.^33,34^ HEK 293 cells were transfected with a plasmid encoding murine CMAH (mCMAH) using a lipid-based transfection reagent. Transfected cells were maintained under selection antibiotic to produce a heterogeneous mix of stable mCMAH-expressing cells (Fig. 2A). Using a non-clonal pool of cells with different levels of CMAH expression ensures that a wide range of Neu5Gc-containing glycoconjugates are represented.

Incorporation of Neu5Gc into cell-surface glycoproteins of mCMAH-HEK cells was validated by flow cytometry using a commercial polyclonal chicken IgY anti-Neu5Gc antibody (Fig. 2B). Neu5Gc presentation on the surface of mCMAH-HEK was also shown by immunostaining with the same anti-Neu5Gc antibody (Fig. S1A, ESI†). The efficient conversion of Neu5Ac into Neu5Gc upon expression of the mCMAH gene was further validated by western blotting (Fig. 2C). Trypsin cleavage of cell surface proteins led to a reduction in total Neu5Gc content of mCMAH-HEK lysates, which confirmed the successful incorporation of synthesised Neu5Gc into glycoproteins (Fig. S1B, ESI†).

Importantly, while stable mCMAH-HEK were used for all subsequent experiments as a matter of convenience, HEK 293 cells transiently transfected with the same murine CMAH construct show no significant difference in Neu5Gc production compared to stably transfected cells (Fig. S1C and D, ESI†), meaning this paired system does not require establishment of a stable CMAH-expressing cell line to be effective.

### Establishing a lysate ELISA method to characterize anti-Neu5Gc antibodies

We next investigated whether lysates from mCMAH- and WT-HEK could be immobilised on a solid support and used as a system to interrogate binding of anti-Neu5Gc antibodies. To achieve this, an indirect ELISA strategy was developed, which is outlined in Fig. 2D. To perform this ‘lysate ELISA’, whole lysates from mCMAH-HEK or WT-HEK cells were directly adsorbed onto 96-well ELISA plates. Plates were blocked with fish gelatin (FG) which does not present Neu5Gc unlike standard blocking agents such as BSA^35^ which show binding to the commercial anti-Neu5Gc IgY (Fig. S2A, ESI†). Anti-Neu5Gc antibodies were added, and binding was detected using an HRP-conjugated secondary antibody. Normalised Neu5Gc-specific signal was calculated as described in the methods section.

Proof of concept experiments with the polyclonal chicken IgY anti-Neu5Gc antibody showed specific IgY binding to mCMAH-HEK lysates (Fig. 2E). We also performed titration experiments using serial dilutions of anti-Neu5Gc IgY spiked into human serum (Fig. 2F). These experiments confirmed the specific detection of anti-Neu5Gc IgY binding even in the presence of a complex matrix such as human serum. The lysate ELISA was also shown to be sensitive, detecting anti-Neu5Gc IgY concentrations as low as 50 ng mL^−1^.

### Affinity purification of anti-Neu5Gc antibodies

While a number of studies have been published using CMAH-positive cells to detect human anti-Neu5Gc antibodies in unprocessed serological samples,^27,36,37^ we were unable to replicate these findings for whole plasma or IVIG using either our lysate ELISA (Fig. S2B, ESI†) or flow cytometry-based approaches (Fig. S2C and D, ESI†). This may be due to high background from the wide range of non-Neu5Gc-containing antigens in the CMAH-HEK and WT-HEK lysates, which could mask the comparatively small pool of these antibodies which are Neu5Gc-reactive. This reinforces the importance of including appropriate controls to identify this background reactivity for each serological specimen being tested, such as paired CMAH-negative cells which will show similar levels of non-Neu5Gc-related antibody binding. As the lysate ELISA and flow cytometry were not able to identify anti-Neu5Gc antibodies in unprocessed plasma, we decided to enrich anti-Neu5Gc antibodies from the serological samples.

Using the strategy outlined in Fig. 2G, serological samples were first pre-cleared on a Neu5Gc-negative column containing HS coupled to resin beads. Flowthrough from the HS column was then loaded onto a Neu5Gc-positive CS column. Increasing concentrations of a simple Neu5Gc α-methyl glycoside (GcOMe) produced in-house, but also commercially available, was then used to elute bound Neu5Gc-specific antibodies. Anti-Neu5Gc antibodies have higher affinity for the α-linked GcOMe than unconjugated Neu5Gc, which exists predominantly in the β-configuration.^38^ However, at concentrations of above 1 mM, unconjugated Neu5Gc also competed for anti-Neu5Gc binding to some extent (Fig. S3, ESI†). It may therefore be possible to use Neu5Gc to elute the antibodies if synthesis or purchase of the methyl glycoside is not accessible.

This purification process was initially trialled on Immunovenin Intact (BulBio NCIPD Ltd, Bulgaria), an IVIG preparation pooled from thousands of healthy donors, which was previously reported to be anti-Neu5Gc-positive on microarray studies.^23^ Fractions of 1 mL were collected, and immunoglobulin-positive fractions were confirmed by western blotting (Fig. S4, ESI†) and pooled. Enriched anti-Neu5Gc antibodies from IVIG (IV-Gc) were tested using the lysate ELISA, but this time with an HRP-conjugated anti-human IgG for detection. Neu5Gc-specific antibody binding was calculated as described in the methods section. IV-Gc preferentially bound to mCMAH-HEK lysates (Fig. 2H, CMAH-WT > 0), which was not observed in the unprocessed IVIG preparation (Fig. S2A, ESI†). In titration experiments, IV-Gc bound to mCMAH-HEK lysates across a range of concentrations (Fig. 2I). Importantly, the interaction between IV-Gc and mCMAH-HEK lysates was shown to be Neu5Gc-dependent, as binding returned to levels of non-transfected cells (CMAH-WT = 0) after co-incubation with a competing dose of GcOMe (Fig. 2J). IV-Gc was also used to adjust the lysate ELISA conditions for efficient detection of enriched human anti-Neu5Gc antibodies, identifying the optimum binding time and plate coating concentration (Fig. S5A and B, ESI†), although this may need to be optimised separately if cells other than HEK cells are used. Specific binding of enriched IV-Gc to mCMAH-HEK was also detectable by flow cytometry and western blotting (Fig. S5C and D, ESI†).

Interestingly, substituting foetal bovine serum (FBS) for CS as a source of Neu5Gc-containing glycans did not allow for the enrichment of Neu5Gc-specific human antibodies (Fig. S6A, ESI†). This may indicate that the similarity of chimpanzee and human glycan profiles^21^ is important to the success of the affinity purification process, or reflect the slightly lower abundance of Neu5Gc in CS compared to FBS (Fig. S6B, ESI†).

**Fig. 3:**
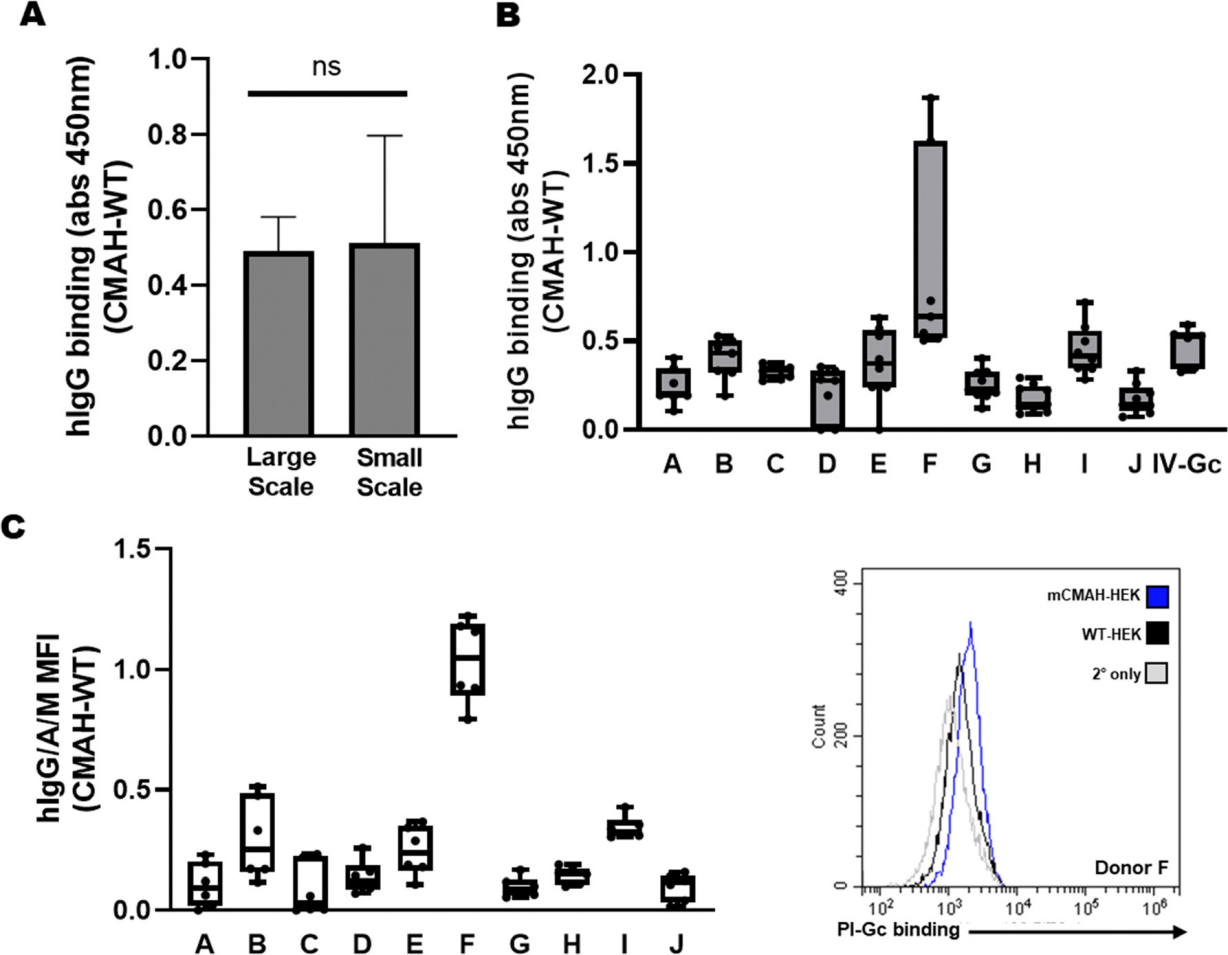
Micro-scale spin columns were used to enrich anti-Neu5Gc antibodies from healthy donor plasma specimens. (A) Testing IV-Gc from gravity columns (large scale) and spin columns (small scale) showed no significant difference in binding to mCMAH-HEK lysates *via* the lysate ELISA. Both IV-Gc preparations were used at 6 μg mL^−1^ (*N* = 3, unpaired *t*-test, *p* = 0.9128). (B) The lysate ELISA was used to record Neu5Gc-specific antibody binding from individual Pl-Gc (donors A to J) and IV-Gc. Pl-Gc and IV-Gc antibodies were applied at a 1 : 25 dilution and detected using an HRP-conjugated anti-human IgG antibody. Data were normalised as described in the methods section. Graph depicts mean ± SD (*N* = 8, 3 independent experiments). (C) Flow cytometry was used to compare Pl-Gc binding to the surface of mCMAH-HEK and WT-HEK cells. Pl-Gc binding was detected using a single FITC-conjugated polyclonal anti-human IgG/A/M antibody. Pl-Gc samples were applied at a 1 : 20 dilution. Graph depicts mean ± SD (*N* = 6, 2 independent experiments). Representative histogram shows Pl-Gc from donor F binding to mCMAH-HEK and WT-HEK cells.

While this large scale affinity purification method effectively enriched Neu5Gc-specific antibodies from IVIG and pooled donor plasma (Fig. S7, ESI†), it required large sample volumes (2–5 mL) and could only process one sample at a time, which is not compatible with enrichment of anti-Neu5Gc antibodies from small volumes of single-donor serological specimens. To address this, the purification process was scaled down to spin columns loaded with HS- and CS-coupled resin, which could process samples of less than 500 μL. IV-Gc prepared using the micro-scale columns showed no significant loss of anti-Neu5Gc reactivity compared to the large-scale columns, highlighting this as a viable scale-down strategy (Fig. 3A).

### Applicability of micro-scale anti-Neu5Gc purification

This approach was then applied to plasma fractions from blood of ten healthy donors of various ages and sexes, to explore whether the affinity purification and lysate ELISA strategy could be utilised to assess individual levels of circulating natural anti-Neu5Gc antibodies. Plasma anti-Neu5Gc antibodies (Pl-Gc) were enriched and tested on the lysate ELISA. Neu5Gc-specific binding was calculated as described in the methods section. This revealed notable differences in Pl-Gc binding to Neu5Gc-containing glycans between the donors (Fig. 3B).

Similar donor variation was detected when measuring Pl-Gc binding to the surface of intact mCMAH-HEK or WT-HEK cells by flow cytometry (Fig. 3C), validating the findings from the lysate ELISA. These experiments using Pl-Gc established the presence of natural human anti-Neu5Gc antibodies in all donors, albeit at low levels in some, and their ability to recognise Neu5Gc-glycoconjugates in their native context on human cells.

**Fig. 4:**
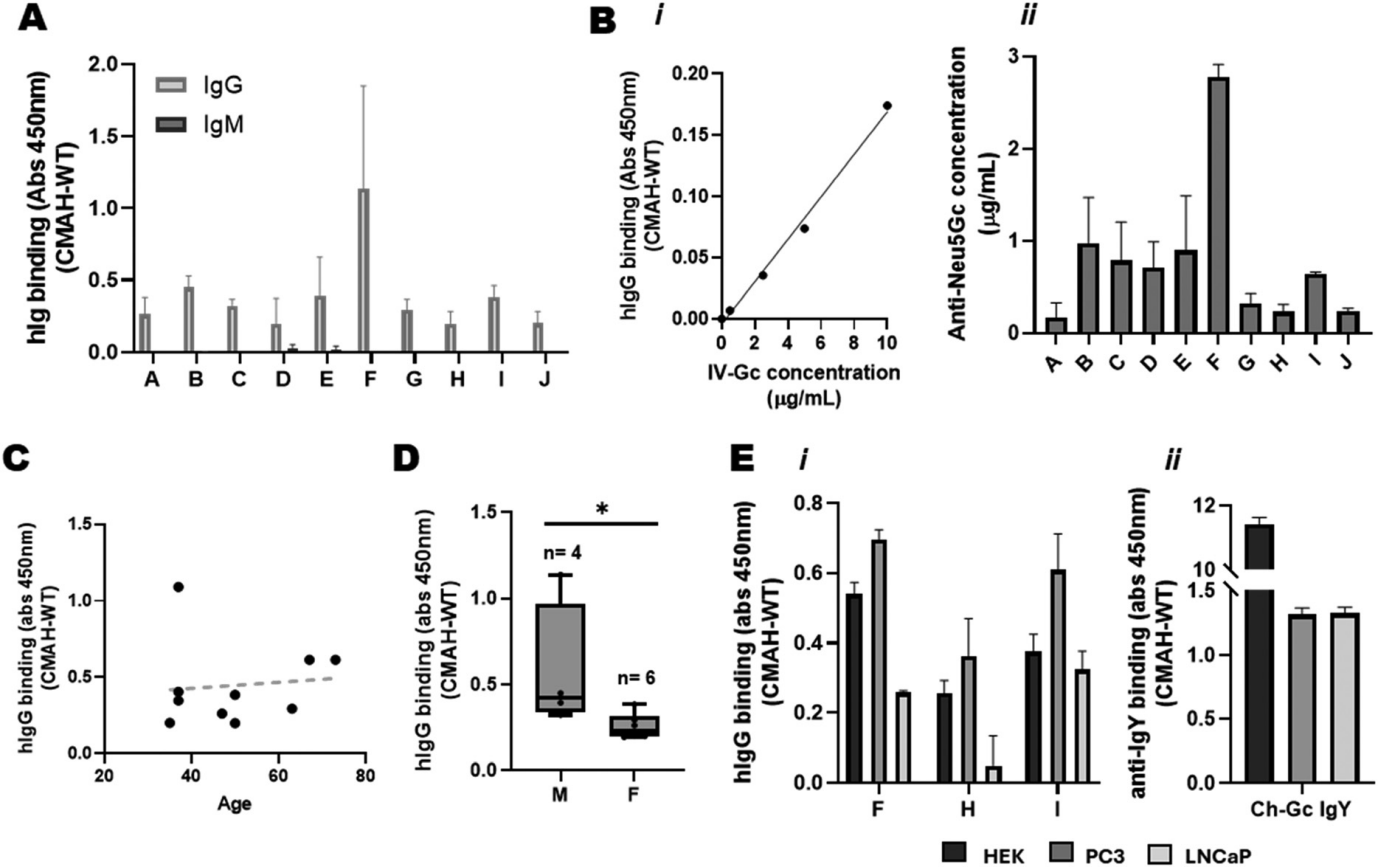
The lysate ELISA strategy can be used for detailed characterisation of the human anti-Neu5Gc antibody response in individual plasma specimens. (A) Isotype-specific Pl-Gc binding was detected on the lysate ELISA. Pl-Gc was added at a 1 : 25 dilution and detected using HRP-conjugated anti-human IgG and IgM antibodies at 1 : 5000 and 1 : 500, respectively. Graph depicts mean ± SD (*N* = 3). (B) The concentration of specific anti-Neu5Gc antibodies enriched from serological samples was estimated using the lysate ELISA. IV-Gc concentration was determined *via* BCA assay, and a titration was used as a standard curve (i) to estimate the concentration of Neu5Gc-specific antibodies in each Pl-Gc specimen (ii). Graph depicts mean ± SD (*N* = 3). (C) Scatterplot showing lack of correlation between anti-Neu5Gc antibody binding and donor age (Pearson's correlation analysis, *R*^2^ = 0.052, *P* = 0.843). Each point depicts mean Neu5Gc-specific abs 450 nm value for one donor (*N* = 6). (D) Graph showing specific anti-Neu5Gc Pl-Gc binding in relation to sex (Mann–Whitney test, *p* = 0.019). Each point depicts the mean Neu5Gc-specific abs 450 nm value for one donor (*N* = 6). (E) Lysate ELISA shows binding of (i) enriched Pl-Gc antibodies from three donors (F, H, I) and (ii) the control chicken anti-Neu5Gc IgY (Ch-Gc IgY) to lysates from mCMAH-HEK or PC3 and LNCaP prostate cancer cell lines transiently transfected with CMAH. Lysates were plated at 2 μg mL^−1^. Results depict mean ± SD (*N* = 3).

Enrichment of anti-Neu5Gc antibodies from donor specimens also presented the opportunity to further dissect different components of the human anti-Neu5Gc antibody response. Use of isotype-specific detection antibodies revealed that the natural anti-Neu5Gc antibody response is predominantly comprised of IgG antibodies, with little to no detection of Neu5Gc-specific IgM antibodies (Fig. 4A), agreeing with previous reports.^27^ This meant that, after assessing IV-Gc concentration using a BCA assay, a titration of IV-Gc could be included as a standard for the lysate ELISA to perform more quantitative analyses (Fig. 4B). This allowed us to estimate the concentration of natural Neu5Gc-specific antibodies enriched from donor specimens at between 0.1 and 2.8 μg mL^−1^.

Individual variation in the anti-Neu5Gc antibody response was not significantly associated with donor age (Fig. 4C). Higher anti-Neu5Gc reactivity was observed in samples from male donors compared to female donors (Fig. 4D). However this is a very small set of samples, and a wider cohort would be needed to ascertain a difference that may be influenced by diet^39^ or immune function^40^ amongst other unaccounted for factors, such as previous infections, or the composition of the microbiome.^22,41^

Additionally, the paired CMAH and WT lysate strategy was not limited to HEK 293 cells. PC3 and LNCaP prostate cancer cell lines could also be transiently transfected with the mCMAH gene and lysates used as the coating antigen for the lysate ELISA. This showed Pl-Gc binding with similar donor variability across the cell lines (Fig. 4Ei). Note that the extent of single donor Pl-Gc binding to lysates from the different cell types did not mirror the binding of the commercial anti-Neu5Gc IgY (Fig. 4Eii). These observations may reflect the variation in glycosylation patterns between the PC3 and LNCaP cell lines,^42^ or recognition of different Neu5Gc-containing glycan structures by antibodies from different donor specimens.

### Exploiting mCMAH-HEK and WT-HEK lysates for micro-scale affinity purification of anti-Neu5Gc antibodies

**Fig. 5:**
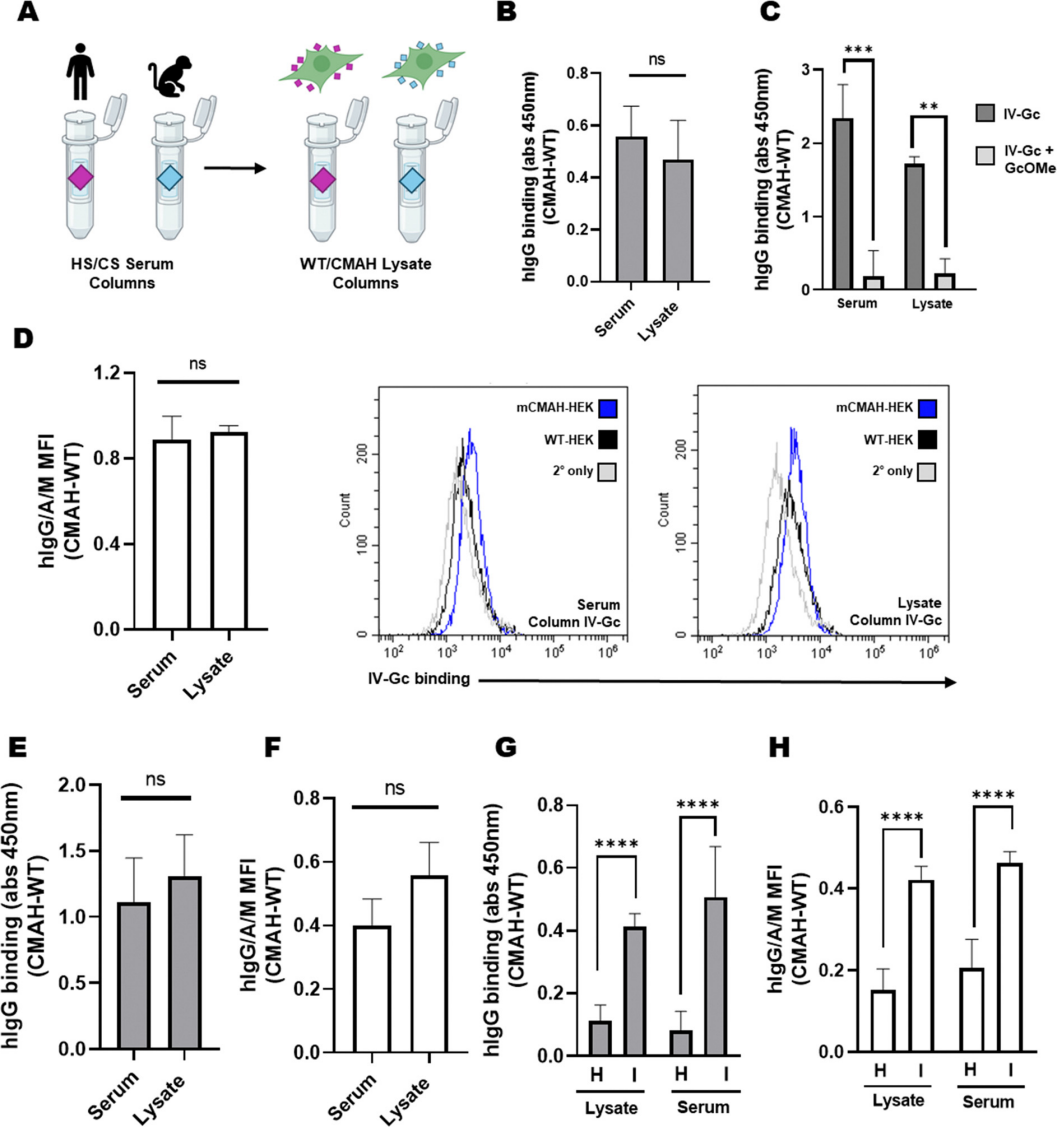
Anti-Neu5Gc antibodies can be enriched using WT-HEK and CMAH-HEK lysate columns. (A) HS and CS can be replaced by WT-HEK and mCMAH-HEK lysates as a source of paired Neu5Gc-negative and Neu5Gc-positive glycans. (B) IV-Gc eluted from the CS/HS columns and WT/CMAH columns show no significant difference in binding to mCMAH-HEK and WT-HEK lysates on the lysate ELISA. Graph depicts mean ± SD (*N* = 3, unpaired *t*-test, *P* = 0.462). (C) Co-incubating IV-Gc from both serum and lysate affinity columns with 2.5 mM GcOMe leads to a reduction in Neu5Gc-specific binding detected by the lysate ELISA (*N* = 3, unpaired *t*-test, ***P = 0.0001, **P = 0.0016). (D) IV-Gc eluted from the serum and lysate columns showed no significant difference in Neu5Gc-specific binding detected by flow cytometry. Graph depicts mean ± SD (*N* = 3, unpaired *t*-test, *P* = 0.618). Representative histograms show anti-human IgG/A/M detection of IV-Gc from serum and lysate columns. (E) Pl-Gc eluted from the serum or lysate columns was tested on the lysate ELISA at a 1 : 10 dilution and detected using an anti-human IgG secondary (*N* = 6, 2 independent experiments, unpaired *t*-test, *P* = 0.3104). (F) Pl-Gc from pooled donor plasma eluted from the serum or lysate columns was tested *via* flow cytometry on mCMAH-HEK or WT-HEK cells. Pl-Gc was used at a 1 : 10 dilution and detected using a FITC-linked anti-human IgG/A/M secondary. Graph depicts mean ± SD (*N* = 3, unpaired *t*-test, *P* = 0.107). Representative histograms show FITC fluorescence for serum column and lysate column Pl-Gc. (G) Binding of Pl-Gc from donors H and I to mCMAH and WT-HEK lysates detected using the lysate ELISA. Pl-Gc was enriched using the lysate column or serum column system and added to the lysate ELISA at a 1 : 10 dilution. Binding was detected using an HRP anti-human IgG antibody. Graph depicts mean ± SD (*N* = 5, two independent experiments, one way ANOVA, *P* = 0.0001) (H) binding of Pl-Gc from donors H and I to mCMAH or WT-HEK was detected by flow cytometry. Pl-Gc was used at a 1 : 10 dilution and detected using a FITC-linked anti-human IgG/A/M secondary. Graph depicts mean ± SD (*N* = 5, two independent experiments, one way ANOVA, *P* = 0.0001).

Our overall aim was to develop an accessible method to enrich anti-Neu5Gc antibodies from serological specimens which can process many samples in parallel. A key limitation to our existing strategy was the cost and amount of human and chimpanzee sera required for preparation of multiple columns. To address this, we explored whether mCMAH-HEK and WT-HEK cell lysates could be used as the paired Neu5Gc-positive and Neu5Gc-negative solid phases micro-scale affinity purification (Fig. 5A). Effective coupling of lysates and presence of Neu5Gc on the resin was confirmed using on-resin BCA and ELISA assays, respectively (Fig. S8A and B, ESI†). Initial tests of this strategy were carried out using IVIG, with anti-Neu5Gc enriched fractions collected from lysate loaded columns (Fig. S8C, ESI†) and tested using the lysate ELISA. We found no significant difference in the detection of Neu5Gc-specific binding for IV-Gc enriched using the lysate or serum affinity columns (Fig. 5B), and a competitive dose of GcOMe equally displaced the binding of IV-Gc enriched from each column system (Fig. 5C). Similarity in Neu5Gc-specific enrichment was also validated by flow cytometry (Fig. 5D). As mentioned previously, using FBS as a source of Neu5Gc-containing glycans instead of CS or mCMAH-HEK lysates was less efficient, suggesting that enriched anti-Neu5Gc antibodies preferentially bind to human or human-like glycan structures, or reflecting a lower concentration of Neu5Gc in FBS (Fig. S8E, ESI†).

This approach was then applied for enrichment of pooled donor plasma specimens. Fractions eluted following the lysate columns (Fig. S8D, ESI†) were similarly enriched in anti-Neu5Gc antibodies than those prepared using the sera columns, detected by both ELISA (Fig. 5E) and flow cytometry (Fig. 5F). Finally, we investigated if this method was sensitive enough to enrich Neu5Gc-specific antibodies from individual donor specimens. Pl-Gc from donors H and I were previously determined to have low and high Neu5Gc-specific binding, respectively. Individual donor (H or I) Pl-Gc enriched using each column system showed similar donor variability when tested by the lysate ELISA (Fig. 5G) and flow cytometry (Fig. 5H). This indicated that the lysate columns are as effective to isolate anti-Neu5Gc antibodies from small volume individual specimens. Importantly, these results also validated the reproducibility of the enrichment and detection strategy, with independent purifications yielding the same Neu5Gc-specific signal for the selected donor specimens.

## Conclusions

Overall, we have optimised a simple method to enrich anti-Neu5Gc antibodies from human serological specimens. This uses a paired column system, with a Neu5Gc-negative ‘pre-cleaning’ column and a Neu5Gc-positive ‘capturing’ column. We have also designed an ELISA method to detect and characterise the binding of resulting enriched anti-Neu5Gc antibodies. This strategy can be applied to small-volume (<500 μL) serological samples, making it compatible with the enrichment of anti-Neu5Gc antibodies from individual donor specimens obtained during routine serological testing. Furthermore, this approach presents Neu5Gc in the context of native human glycan structures without requiring complex chemical or chemoenzymatic synthesis, and can be performed using commercially available reagents. Use of cell lysates from WT and CMAH-transfected human cells also avoids the expense of large volumes of sera for column preparation, further increasing its accessibility.

Additionally, the lysate ELISA detection approach could be modified for use on various human cell lines. This presents the valuable opportunity to explore the anti-Neu5Gc antibody response against specific tissue types. Considering biological context is important when studying anti-Neu5Gc antibodies, given the marked variation in glycosylation patterns between different human tissues and disease states,^43^ and the potential that different individuals may express anti-Neu5Gc antibodies against a different profile of glycan structures.^44^ We also anticipate that this method may be adapted to enrich antibodies from individual donor specimens against other immunogenic glycans, for example αGal, which has been implicated in xenograft rejection and red meat allergy.^45,46^

Finally, by applying this enrichment and detection workflow to healthy donor plasma specimens, we revealed that anti-Neu5Gc antibodies could be reproducibly detected across a group of ten healthy donors of different ages and sexes. Anti-Neu5Gc antibodies were detectable in all donors regardless of age or sex, and exhibited individual differences which may be influenced by factors such as diet or past infections.^9,12,47^ Importantly, even in donor specimens with relatively low anti-Neu5Gc reactivity, these antibodies could bind to Neu5Gc-containing glycans on the surface of intact human cells, supporting the hypothesis that they may be relevant to human health.

## Materials and methods

### Reagents and antibodies

All materials and reagents were purchased from Thermo Fisher Scientific and chemicals sourced from Merck, unless otherwise stated. The purified chicken polyclonal IgY anti-Neu5Gc antibody (Poly21469) was purchased from BioLegend, secondary anti-chicken or anti-human antibodies (HRP- or Alexa-488) were purchased from Invitrogen. Commercial human serum was purchased from Sigma Aldrich.

### Cell culture

HEK 293 cells were grown in Dulbecco's modified Eagle medium supplemented with 10% FBS, 10 000 units per ml penicillin, 10 000 μg ml^−1^ streptomycin and 2 mM l-glutamine. HEK 293 stable transfectants were maintained in this same medium with addition of 125 μg mL^−1^ hygromycin. All cells were maintained in T75 tissue culture flasks at 37 °C under 5% CO_2_ and were passaged upon reaching confluence. Cells were detached using trypsin/EDTA or 10 mM EDTA-PBS for passage or experiments, respectively.

### Stable transfection with mCMAH cDNA

Mouse CMAH (mCMAH) cDNA in a pCMV3 expression vector was purchased from Sino Biological (#NM_001111110.2). HEK 293 cells were transfected with 1 μg mL^−1^ cDNA using the XtremeGENE HP transfection reagent (Roche, #6366244001). Stably transfected cells were selected using 125 μg mL^−1^ hygromycin and maintained as a heterogeneous pool. LNCaP and PC3 cells were transiently transfected with 2 μg mL^−1^ mCMAH cDNA using the same transfection reagent. Transiently transfected cells were harvested 48 h after transfection.

### IVIG and plasma samples

ImmunoVenin Intact IVIG was a generous gift from BulBio NCIPD Ltd, Bulgaria. Frozen plasma fractions used were isolated from blood samples of healthy volunteers collected following written informed consent, according to a blood donation protocol from the University of York approved by the Biology department Ethics Committee (BEC: PBMC donation protocol Version 3; November 2019) and delivered in compliance with ICH GCP, the Data Protection Act, and other regulatory requirements, as appropriate. Additional ethical approval was received from the Health Research Authority through the Yorkshire and Humber research ethics committee (REC reference 19/YH/0394, for IRAS project ID: 269597).^48^

### Preparation of cell lysates

Cells were harvested using 10 mM EDTA in PBS. Lysis was carried out using 1% Triton X-100 lysis buffer (0.1 M Tris pH 8, 0.1 M NaCl, 1 mM CaCl_2_, 1% Triton X-100) supplemented with a protease inhibitor cocktail (Roche, #11697498001) for 15 min. Lysates were clarified by centrifugation for 30 min, 17 000×*g* at 4 °C. Protein concentration was determined *via* BCA assay.

### SDS-PAGE and western blotting

Samples were boiled at 95 °C in SDS sample buffer (2% SDS, 2 mM β-mercaptoethanol, 4% glycerol, 0.01% bromophenol blue, 0.04 M Tris-HCl pH 6.8) for 5 min, then loaded onto 10% acrylamide SDS-PAGE gels. Gels were run at 150 V until fully resolved. Gels were stained with Coomassie blue, destained, and imaged using an iBright 1500 imaging system (Invitrogen, #A44114). For western blotting, proteins were transferred onto nitrocellulose using the Trans-Blot Turbo transfer system (Bio-Rad, #1704150). Membranes were blocked in 1% fish gelatin in PBS plus 0.05% TWEEN-20 (FG/PBST). Chicken anti-Neu5Gc IgY (Biolegend, #146903) at a 1 : 4000 dilution was added in FG/PBST overnight at 4 °C. HRP anti-chicken IgY (Invitrogen, #A16054) secondary antibody was added at 1 : 500 in FG/PBST for 1 h at room temperature. Membranes were developed using BM Chemiluminescence western blotting substrate (Roche, #11500708001) and images were acquired using the iBright 1500 imaging system.

### Lysate ELISA

Flat bottom NUNC Maxisorp 96-well plates were coated overnight with 1 μg mL^−1^ mCMAH-HEK or WT-HEK lysates in 0.05 M carbonate coating buffer (pH 9.6). Wells were blocked with 1% fish gelatin in PBS (FG/PBS) for 2 h at room temperature. Anti-chicken IgY was added at 1 : 5000 in FG/PBS and incubated for 1 h at room temperature. Affinity purified antibodies were added 1 : 25 in FG/PBS and incubated for 3 h at room temperature. Wells were washed 3× with 0.05% PBST and HRP anti-chicken IgY (Invitrogen, #A16054, 1 : 1000), HRP-anti-human IgG (FineTest, #FNSA-0105, 1 : 5000) or HRP-anti-human IgM (FineTest, #FNSA-0103, 1 : 500) was added in PBS and incubated for 1 h at RT. Plates were washed 3× with PBST and developed using TMB high sensitivity substrate (BioLegend, #421501). The reaction was stopped using 1 M H_2_SO_4_ and absorbance was read at 450 nm.

Date were normalised by dividing all raw Abs 450 nm values by the Abs 450 nm value for the secondary antibody only, in order to account for differences in overall absorbance reading between experiments. To calculate Neu5Gc-specific antibody binding, normalised Abs 450 nm values for WT-HEK Neu5Gc-negative were subtracted from normalised Abs 450 nm values for mCMAH-HEK Neu5Gc-positive wells.Neu5Gc specific Abs 450 nm = (mCMAH Abs 450 nm/2° only Abs 450 nm) − (WT Abs 450 nm/2° only Abs 450 nm)

### ELISA detection of Neu5Gc content in human and chimpanzee sera

Flat bottom NUNC Maxisorp 96-well plates were coated overnight with pooled human serum (Sigma, #H6914) or chimpanzee serum (https://Antibodies.com, #A116435) at a 1 : 100 dilution in 0.05 M carbonate coating buffer (pH 9.6). Wells were blocked with 1% fish gelatin in PBS (FG/PBS) for 2 h at room temperature. HRP-conjugated anti-chicken IgY was added at 1 : 1000 in FG/PBS and incubated for 1 h at room temperature. Wells were washed 3× with 0.05% PBST and HRP anti-chicken IgY (Invitrogen, #A16054, 1 : 1000) was added in PBS and incubated for 1 h at RT. Plates were washed 3× with PBST and developed using TMB high sensitivity substrate (BioLegend, #421501). The reaction was stopped using 1 M H_2_SO_4_ and absorbance was read at 450 nm.

### Flow cytometry assays

Cells detached in PBS/EDTA were harvested and resuspended in FACS buffer (PBS, 1% FBS, 0.05% sodium azide). Cells were transferred to 96-well round bottom plates (100 000 cells per well) and kept on ice. Plated cells were washed through addition of ice-cold FACS buffer followed by a 5 min 1500×*g* spin at 4 °C. After three washes, Fc receptors were blocked using 30 μg mL^−1^ rat IgG in FACS buffer for 30 min, and then incubated with polyclonal chicken anti-Neu5Gc IgY (1 : 1000) or affinity purified human anti-Neu5Gc fractions (1 : 20) in FACS buffer for 4 h under agitation at 4 °C. After three more washes, Alexa-488 anti-chicken IgY (Invitrogen, #A-11039, 1 : 200) or FITC anti-human IgG/A/M (Invitrogen, #15440814, 1 : 200) secondary antibodies diluted in FACS buffer were added for 1 h. Cells were washed three times and fixed in 1% formaldehyde containing FACS buffer overnight at 4 °C. Washed, fixed cells were resuspended in FACS buffer and samples run on a CytoFLEX S Flow cytometer (Beckman Coulter) recording readings of Alexa-488 mean fluorescence intensity (MFI).

Normalised MFI was calculated by subtracting cell-only MFI values. Specific MFI was then calculated by dividing normalised MFI for specimen anti-Neu5Gc antibodies by secondary only MFI values to account for differences in overall MFI value between experiments. Neu5Gc-specific MFI was then calculated as WT-HEK MFI values subtracted from mCMAH-HEK MFI values.MFI = Sample mean fluorescence − Cell only mean fluorescenceNeu5Gc-specific MFI = (mCMAH MFI/2° only MFI) − (WT MFI/2° only MFI)

### Neu5Gc-α-methyl glycoside synthesis

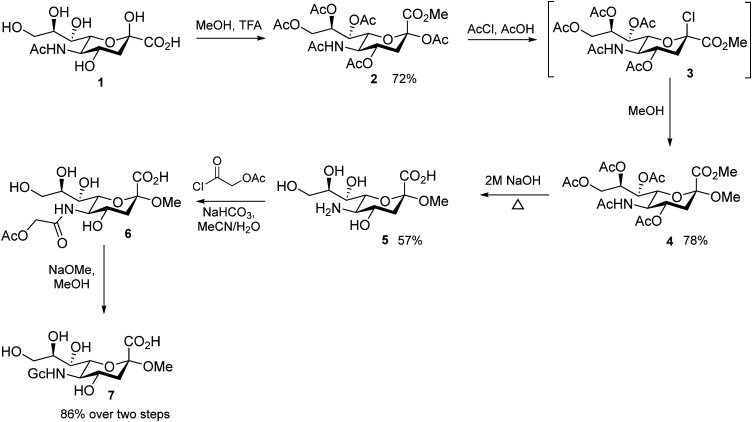



Neu5Gc α-methyl glycoside (GcOMe) was prepared according to published protocols.^23,44,49^ Detailed experimental information can be found in Scheme S1 (ESI†).

### Micro-scale affinity purification of anti-Neu5Gc antibodies

To prepare serum-loaded columns, heat inactivated HS and CS in 0.1 M 3-(*N*-morpholino)propanesulfonic acid (MOPS) buffer (pH 7.5) were coupled to Affi-gel 15 resin (Bio-Rad, #153-6052) according to the manufacturer's protocol. Beads were loaded with a final concentration of 1 mg serum per 1 mL resin. To prepare lysate-loaded columns, lysates were prepared as described above and coupled to Affi-gel 15 resin at 250 μg lysate per mL of resin in MOPS buffer. Coupling was carried out overnight at 4 °C.

Active esters in the resin were neutralised with 0.1 M ethanolamine-HCl for 1 h. Into each empty mini Bio-spin column (BioRad, #7326207) 1 mL resin was loaded to form Neu5Gc-negative (HS or WT-HEK lysate) columns and Neu5Gc-positive (CS or mCMAH-HEK lysate) columns. To prime the columns, 3× washes with 50 mM sodium acetate (pH 5.5), 3× washes with PBS (pH 7.4), 3× washes with 0.1 M citric acid (pH 3.0) and 3× washes with PBS were carried out sequentially, each for 1 min at 500 rpm. Columns were stored in PBS containing 0.5% sodium azide.

Plasma samples were heat inactivated for 30 minutes at 56 °C and diluted in PBS to a final volume of 500 μL. Plasma was loaded onto the primed HS columns and incubated for 5 min, before centrifuging for 3 min at 500 rpm. The flowthrough was loaded back onto the HS column for a total of 4 rounds of pre-clearing. The resulting flowthrough was then loaded onto the CS column and incubated for 3 min, followed by 3 min centrifugation. The flowthrough was re-loaded through the column for a total of 5 passes. To elute non-specifically bound antibodies, the Neu5Gc-positive columns were washed with 5 mM glucuronic acid for 1 min at 500 rpm, then washed with PBS. To elute anti-Neu5Gc antibodies, 400 μL 0.5 mM Neu5Gc α-methyl glycoside (GcOMe) in PBS was added to the columns and incubated for 6 min, followed by 3 min centrifugation at 500 rpm. The previous step was repeated with 400 μL 2.5 mM GcOMe. To elute additional antibodies, the columns were washed with citric acid for 1 min at 500 rpm and the flowthrough was neutralised with Tris-HCl (pH 9). Antibodies were dialysed against PBS overnight through a 3500 MWCO membrane and stored at −20 °C. For experiments, the 0.5 mM and 2.5 mM elutions were pooled 1 : 1.

## Data availability

The data supporting this article have been included as part of the ESI.†

## Conflicts of interest

There are no conflicts to declare.

## Supplementary Material

CB-OLF-D5CB00073D-s001
