# Supplementary material for: A general and accessible approach to enrichment and characterisation of natural anti-Neu5Gc antibodies from human samples
Source: RSC Chem Biol. 2025 May 15;6(7):1135–47. doi: 10.1039/d5cb00073d (PMC12100518; doi:10.1039/d5cb00073d)
Supplement: CB-006-D5CB00073D-s001 [file CB-006-D5CB00073D-s001.pdf]

Supplementary Information (SI) for RSC Chemical Biology.
This journal is © The Royal Society of Chemistry 2025

A general and accessible approach to enrichment and characterisation of natural anti-Neu5Gc antibodies from human samples.

Esme Hutton[1], Yumiko Uno[1,2], Emma Scott[3], Craig Robson[3], Martin Fascione[1*], Nathalie Signoret[2*]

[1] *Department of Chemistry, University of York, York, United Kingdom*

[2] *Hull York Medical School, University of York, York, United Kingdom*

[3] *Newcastle University, Centre for Cancer, Newcastle, United Kingdom*

* Corresponding authors (nathalie.signoret@york.ac.uk, martin.fascione@york.ac.uk)

SUPPORTING INFORMATION

Supplementary methods

Chemical synthesis of Neu5Gc α-methyl glycoside

Immunofluorescence staining

Supplementary figures

Figure S1: mCMAH-transfected HEK 293 cells incorporate Neu5Gc into cell surface glycoproteins.

Figure S2: Anti-Neu5Gc antibodies in whole plasma or IVIG could not be detected by lysate ELISA or flow cytometry.

Figure S3: both the Neu5Gc α-methyl glycoside (GcOMe) and Neu5Gc can compete with mCMAH-HEK lysates for anti-Neu5Gc IgY binding.

Figure S4: Fractions from the affinity purification process on IVIG detected by western blotting

Figure S5: Application of enriched anti-Neu5Gc antibodies from IVIG (IV-Gc) to the lysate ELISA, flow cytometry and western blotting.

Figure S6: Chimpanzee serum cannot be replaced by FBS in the paired column method to enrich anti-Neu5Gc antibodies.

Figure S7: Large-scale affinity purification can also be used to isolate anti-Neu5Gc antibodies (PI-Gc) from pooled human plasma.

Figure S8: Preparation of and enrichment of anti-Neu5Gc antibodies using mCMAH-HEK and WT-HEK lysate columns.

Supplementary methods

Chemical synthesis of Neu5Gc α-methyl glycoside

2-*O*-Methyl-5-*N*-glycolyl-α-D-neuraminic acid (**7**) is commercially available (CAS: 14206-42-9), but here we also report a reproducible gram scale synthesis (**Scheme S1**).

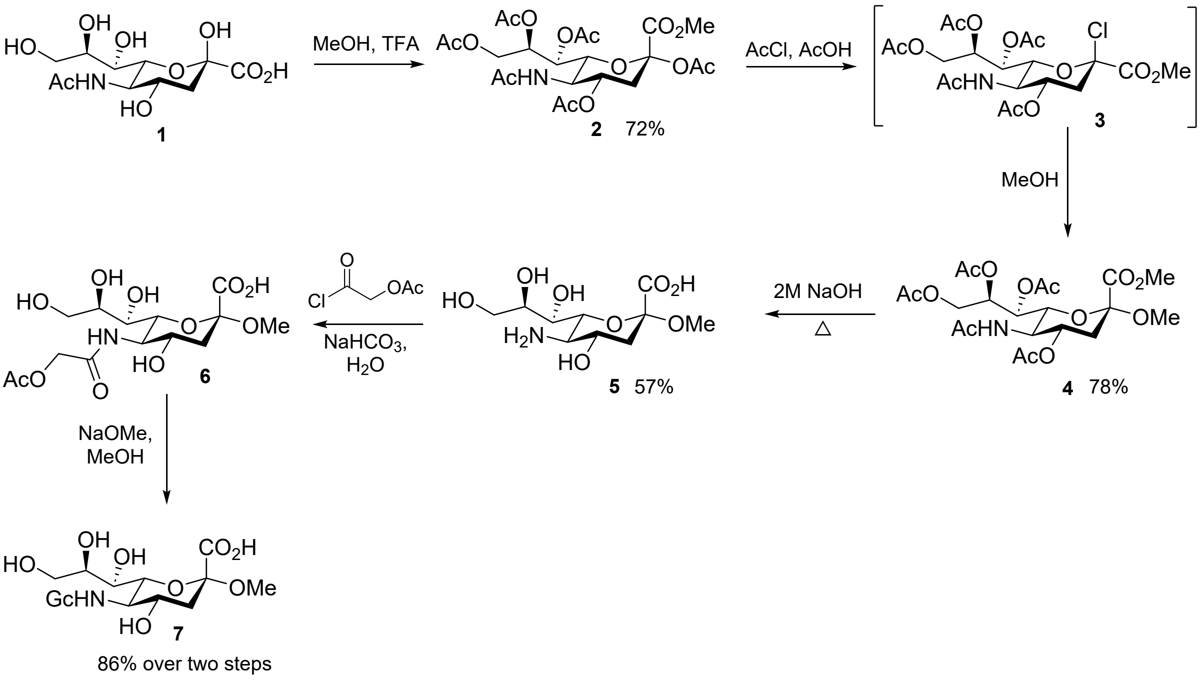

Scheme S1: Overall synthetic strategy for synthesis of Neu5Gc-α-OMe **7**, from Neu5Ac **1**.

2,4,7,8,9-Penta-*O*-acetyl-5-*N*-acetyl-α-D-neuraminic acid methyl ester (2)

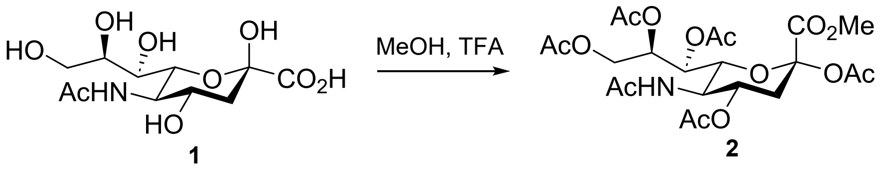

Neu5Ac **1** (5 g, 16 mmol) was suspended in methanol (100 mL) before the addition of trifluoracetic acid (400 μL), the resultant cloudy solution was then stirred under N_2 (g) for 24 h before further addition of trifluoracetic acid (100 μL) and stirring for 36 h in total, until TLC (35% MeOH/DCM) indicated the reaction had proceeded to completion. The resulting cloudy solution was filtered through celite and concentrated under reduced pressure affording **2** (3.54 g, 72%) as a colourless solid, which was used in the next step without further purification.

2-*O*-Methyl-4,7,8,9-tetra-*O*-acetyl-5-*N*-acetyl-α-D-neuraminic acid methyl ester (4)

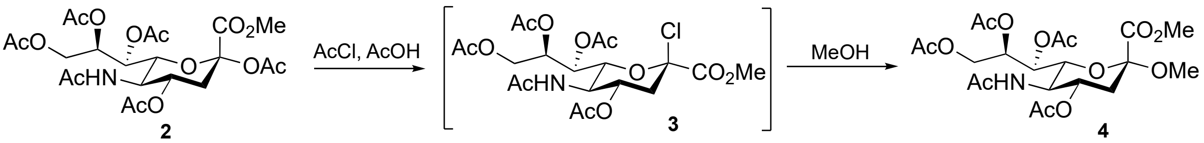

2 (3.6 g, 11 mmol) was dissolved in acetic acid (43 mL) and cooled on ice before slow addition of acetyl chloride (79 mL, 59 mmol) under N_2 (g), the resultant solution was allowed to warm to rt then stirred for 72 h. The solution was then concentrated under reduced pressure affording crude **3** which was redissolved in anhydrous MeOH (40 mL) and stirred under N_2 (g) for 1 h. The solution was then concentrated under reduced pressure, resuspended in EtOAc (70 mL) then washed with aq. $NaHCO_3$ (50 mL) and aq. NaCl (50 mL) and the organic layer dried ($MgSO_4$) and concentrated under reduced pressure to afford a crude brown solid. The solid was purified by flash silica column chromatography (20% EtOAc/Hexane to 70% EtOAc/Hexane) to afford **4** (2.8 g, 78%) as a colourless solid, with characterisation as previously reported in the literature **[Kononov *et al.* Carbohydrate Chemistry: Proven Synthetic Methods, Vol. 2. 2014. p. 197-206]**

δ_H (400 MHz, $CDCl_3$); 5.41 (ddd, 1H, *J* 8.4 Hz, *J* 5.6 Hz, *J* 2.7 Hz, H-8), 5.32 (dd, 1H, *J* 8.5 Hz, *J* 2.1 Hz, H-6), 5.23 (d, 1H, *J* 9.7 Hz, NH), 4.84 (ddd, 1H, *J* 12.4 Hz, *J* 9.5 Hz, *J* 4.6 Hz, H-4), 4.31 (dd, 1H, *J* 12.5 Hz, *J* 2.8 Hz, H-9), 4.14-4.08 (m, 2H, H-7, H-9'), 3.86 (m, 1H, H-5), 3.80 (s, 3H, $COOCH_3$), 3.31 (s, 3H, OCH_3), 2.56 (dd, 1H, *J* 12.8 Hz, *J* 4.6 Hz, H-3_{eq}), 2.15 (s, 3H, OAc), 2.13 (s, 3H, OAc), 2.03 (s, 3H, OAc), 2.02 (s, 3H, OAc), 1.93 (dd, 1H, *J* 12.8 Hz, *J* 9.5 Hz, H-3_{ax}), 1.87 (s, 3H, NHAc)

δ_C (50 MHz, $CDCl_3$); 171.6, 171.5, 171.1, 170.6, 170.5 (C=O), 168.6 (C-1), 99.4 (C-2), 72.9 (C-6), 69.5 (C-4), 67.7 (C-8), 62.8 (C-7), 60.9 (C-9), 53.2 ($CO_2\underline{C}H_3$), 52.9 (OCH_3), 49.9 (C-5), 38.3 (C-3), 23.6 ($NC(O)CH_3$), 21.6, 21.5, 21.3, 21.2 (OAc)

2-*O*-Methyl-α-D-neuraminic acid (5)

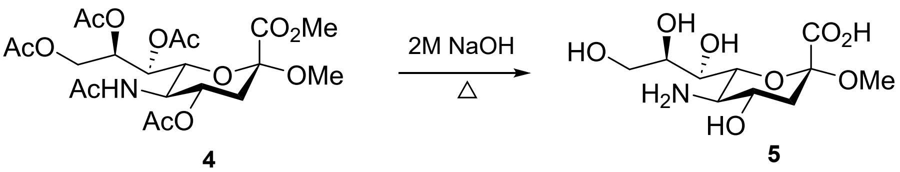

4 (1.8 g, 3.6 mmol) was suspended in 2M NaOH (13 mL) and refluxed for 18 h, the resultant solution was allowed to cool to rt before neutralising with amberlite IRC-120 H^+ resin, filtering and partially concentrated under reduced pressure before lyophilisation to afford **5** (1.605 g, 57%) as a brown solid, which was used in the next step without further purification.

2-*O*-Methyl-5-*N*-glycolyl-α-D-neuraminic acid (7)

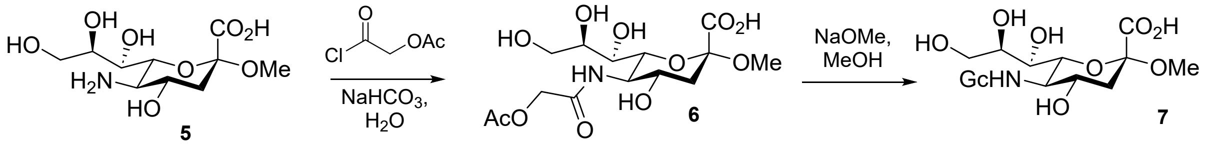

5 (0.87 g, 3.6 mmol) was dissolved in a solution of $NaHCO_3$ (3.02 g, 36 mmol, 10 equiv.) in H_2O and cooled to 0 °C on ice before dropwise addition of acetoxyacetyl chloride (3.48 mL, 36 mmol, 10 equiv.) with stirring. The solution was then allowed to warm to rt over 16 h before neutralising with amberlite IRC-120 H^+ resin, filtered and partially concentrated under reduced pressure before lyophilisation to afford crude **6** as a syrup. The syrup was then redissolved in anhydrous MeOH (10 mL) before the addition of 0.5 M NaOMe in MeOH (5 mL) and stirred for 2 h until completion. The solution was then neutralised using amberlite IRC-120 H^+ resin, filtered and concentrated under reduced pressure to afford Neu5Gc α-methyl glycoside **7** (0.9 g, 86% over two steps) as an orange solid, with characterisation as previously reported in the literature. **[Chopra P. *et al*, Tetrahedron Letters. 2013;54(41):5558-61.], [Padler-Karavani V. *et al*, Glycobiology. 2008;18(10):818-30.]**

Neu5Gc α-methyl glycoside **7** can be further purified using anion exchange chromatography, however crude **7** is of sufficient purity for elution of anti-Neu5Gc antibodies.

δ_H (400 MHz, D_2O); 4.11 (s, 2H, COC$\underline{H}_2$OH), 3.91-3.75 (m, 5H, H-4, H-5, H-6, H-8, H-9), 3.68-3.55 (m, 2H, H-7, H-9'), 3.34 (s, 3H, OCH_3), 2.73 (dd, 1H, *J* 12.5 Hz, *J* 4.9 Hz, H-3_{eq}), 1.64 (app t, 1H, *J* 12.1 Hz, *J* 13.4 Hz, H-3_{ax})

δ_C (50 MHz, D_2O); 179.4 (NHCO, C-1), 100.7 (C-2), 72.5 (C-6), 71.7 (C-8), 68.2 (C-7), 68.0 (C-4), 62.7 (C-9), 60.9 (CH_2OH), 51.6 (OMe, C-5), 40.2 (C-3)

Immunofluorescence staining

mCMAH-HEK and WT-HEK cells were grown on coverslips. Cells were washed 2x with PBS and fixed with 3% formaldehyde in PBS for 15 minutes at RT. After 2 PBS washes, free aldehydes were quenched with 50 mM ammonium chloride for 20 min. Cells were washed 2x with PBS, then blocked with 1% human serum in PBS (HS/PBS) for 30 min at RT. Anti-Neu5Gc IgY was added at 1:1000 in 1% HS/PBS for 1h at RT. Cells were washed 3x with PBS for 5 min each, then Alexa-488 anti-chicken IgY (Invitrogen, #A-11039) was added at 1:500 in PBS for 1h at RT. After 3x 5 min PBS washes, coverslips were mounted using Mowiol containing DAPI at 1:5000. Coverslips were imaged using an LSM980 upright confocal microscope at the University of York technology facility. Images were taken at equal laser intensity for WT-HEK and mCMAH-HEK and processed using Image J.

Supplemenatry figures

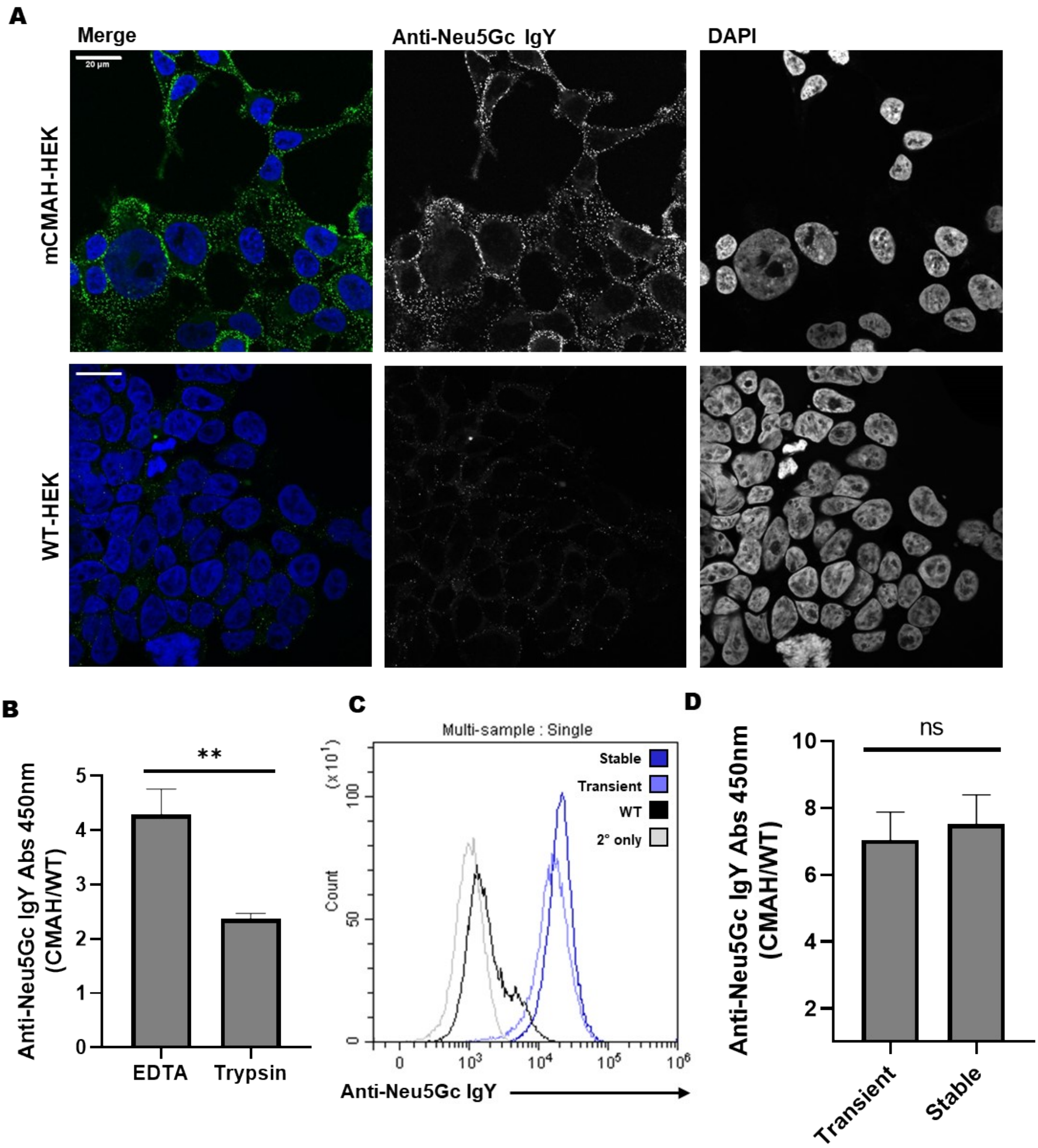

Figure S1: ***mCMAH-transfected HEK 293 cells incorporate Neu5Gc into cell surface glycoproteins. (A)*** *Representative confocal Z-stacks for anti-Neu5Gc immunostaining on mCMAH-HEK and WT-HEK cells. Cells were fixed in 3% formaldehyde and stained using anti-Neu5Gc IgY and an alexa-488 anti-chicken IgY secondary. Images were taken using the same laser settings and images were adjusted to equal intensity for each cell type. Scale bars indicate 20 µm.* ***(B)*** *Harvesting cells with trypsin prior to lysis led to a decrease in anti-Neu5Gc IgY antibody binding to mCMAH-HEK lysates detected by ELISA. Neu5Gc loss was not seen when cells were mechanically harvested using 10 mM EDTA in PBS. (N=4, 2 independent experiments, unpaired t-test, p= 0.0029)* ***(C+D)*** *There is no significant difference in Neu5Gc content of transiently and stably transfected CMAH-positive HEK cells. Neu5Gc was detected*

on whole cells by flow cytometry ***(D)*** *or cell lysates by ELISA* ***(E)*** *using the anti-Neu5Gc IgY antibody followed by Alexa-488 conjugated or HRP conjugated anti-chicken IgY. (Unpaired t-test, p= 0.4301).*

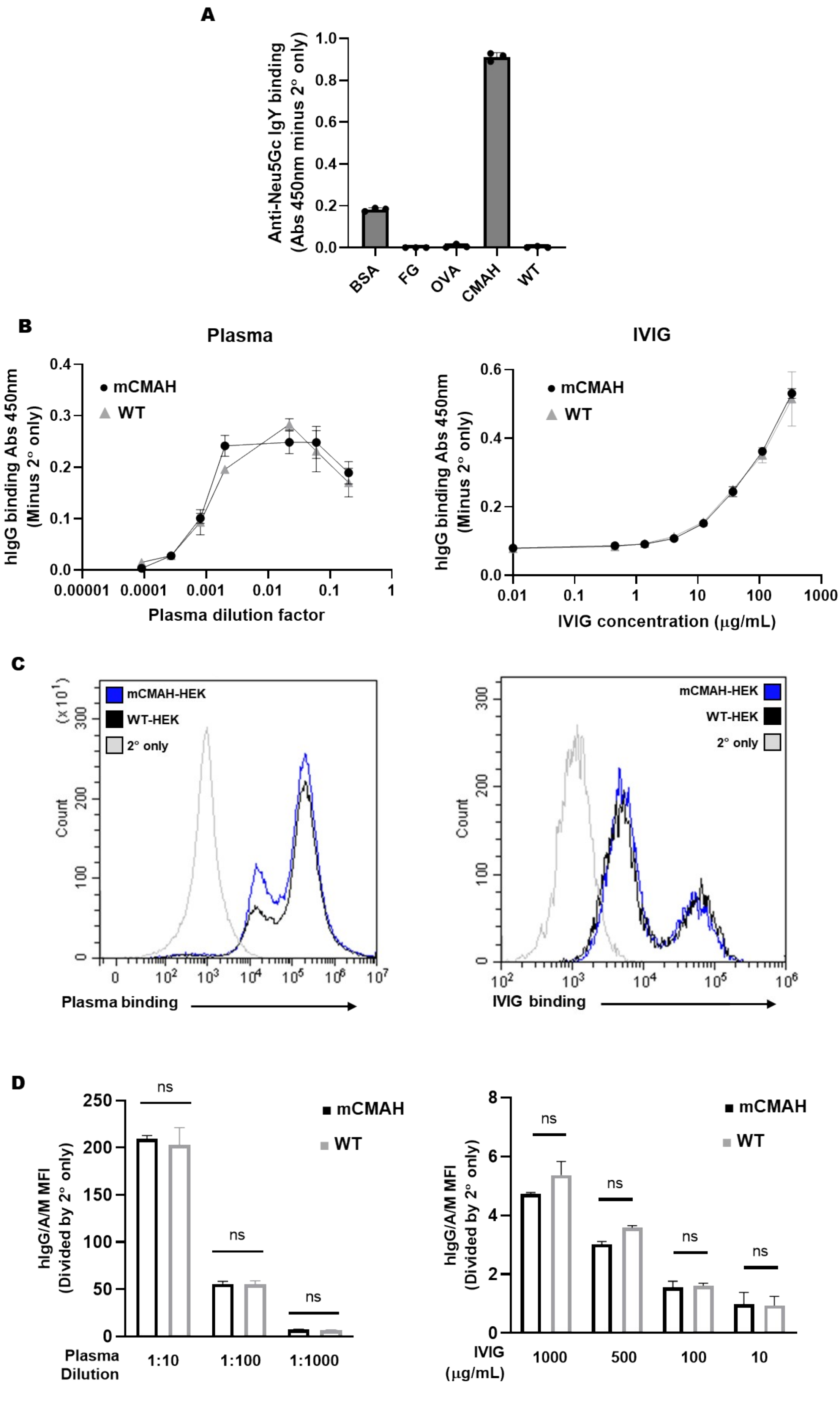
A
Anti-Neu5Gc IgY binding (Abs 450nm minus 2° only)
1.0
0.8
0.6
0.4
0.2
0.0
BSA
FG
OVA
CMAH
WT
B
Plasma
hIgG binding Abs 450nm (Minus 2° only)
mCMAH
WT
0.00001
0.0001
0.001
0.01
0.1
1
Plasma dilution factor
IVIG
0.01
0.1
1
10
100
1000
IVIG concentration (μg/mL)
C
mCMAH-HEK
WT-HEK
2° only
Count
Plasma binding
IVIG binding
D
hIgG/A/M MFI (Divided by 2° only)
ns
mCMAH
WT
250
200
150
100
50
0
Plasma Dilution
1:10
1:100
1:1000
IVIG (μg/mL)
1000
500
100
10

Figure S2: ***Anti-Neu5Gc antibodies in whole plasma or IVIG could not be detected by lysate ELISA or flow cytometry.*** ***(A)*** *Anti-Neu5Gc IgY binding to ELISA plates coated with bovine serum albumin (BSA), fish gelatin (FG), ovalbumin (OVA), mCMAH-HEK lysates (CMAH) and WT-HEK lysates (WT) all at 1 ug/mL. Antibody binding was detected using an HRP-conjugated anti-chicken IgY secondary. (N=3, 1 independent experiment).* ***(B)*** *Titrations of plasma pooled from several healthy donors or IVIG were added to the lysate ELISA in PBS. Antibody binding was detected using an HRP-anti-human IgG antibody. (N=3, 1 independent experiment)* ***(C)*** *Representative histogram overlays showing whole plasma or IVIG at a 1:100 dilution binding to mCMAH-HEK and WT-HEK cells by flow cytometry. Antibody binding was detected using a FITC-conjugated anti-human IgG/A/M secondary.* ***(D)*** *Quantification of plasma and IVIG binding to mCMAH-HEK and WT-HEK cells detected by flow cytometry using a FITC-anti-human IgG/A/M antibody. (N=3, 1 independent experiment, one way ANOVA, p= 0. 0.9225).*

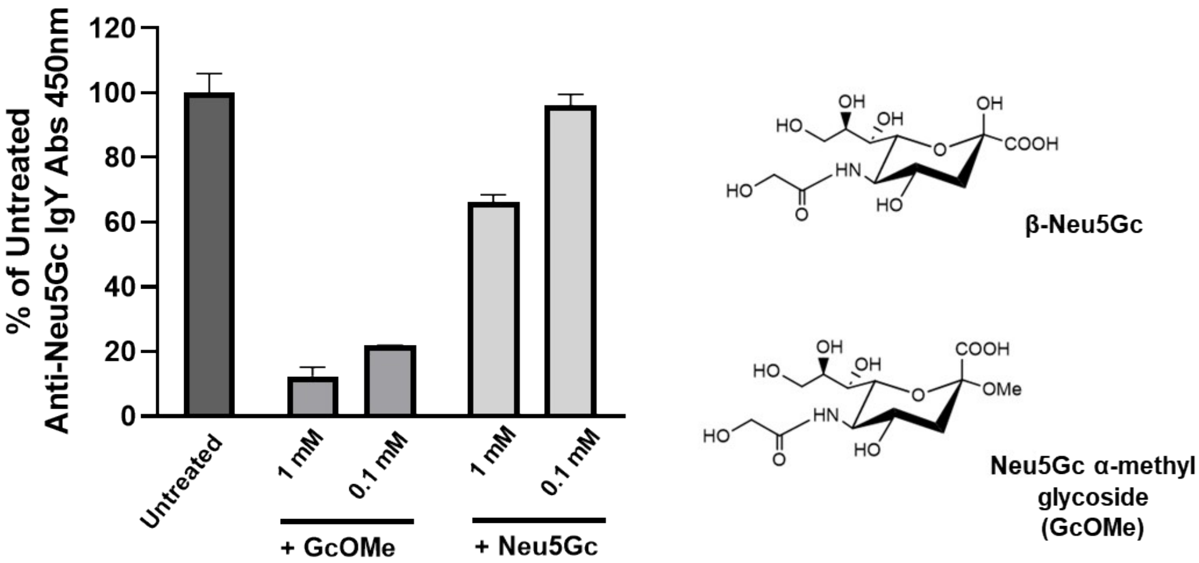

Figure S3: ***Both the Neu5Gc α-methyl glycoside (GcOMe) and free Neu5Gc can compete with mCMAH-HEK lysates for anti-Neu5Gc IgY binding.*** *Anti-Neu5Gc IgY was co-incubated with GcOMe or Neu5Gc at 1 mM or 0.1 mM for 2h at 4°C. Binding to mCMAH-HEK or WT-HEK lysates was detected using an HRP-anti-chicken IgY antibody. For co-incubation conditions, the secondary-only wells were also treated with GcOMe or Neu5Gc. The decrease in anti-Neu5Gc IgY Abs 450nm was calculated as a percentage of anti-Neu5Gc IgY not co-incubated with GcOMe / Neu5Gc. (N=3, 1 independent experiment.).*

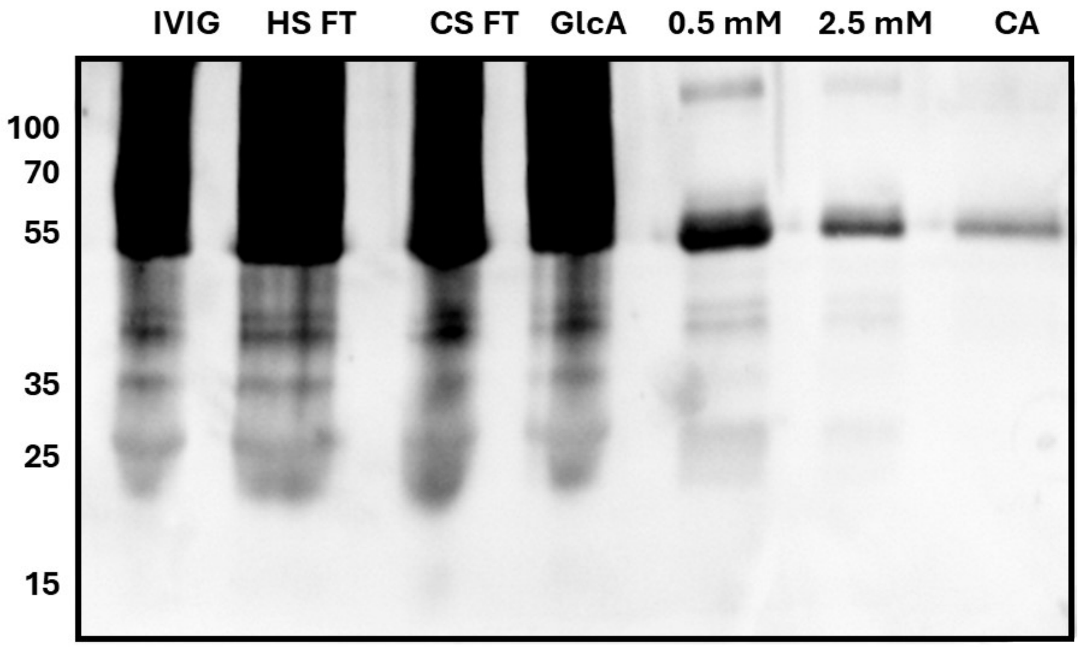

Figure S4: ***Fractions from the affinity purification of IVIG, detected by western blotting.*** *For each fraction, 30 µL was loaded into the lanes of a 10% acrylamide SDS-PAGE gel. Proteins were transferred to a nitrocellulose membrane and detected using an HRP-anti-human IgG antibody. From left to right: Original IVIG sample, flowthrough from HS column (pre-absorbed sample), flowthrough from CS column (non-anti-Neu5Gc antibodies), Glucuronic acid (GlcA) elution (nonspecifically bound antibodies), 0.5 mM GcOMe elution, 2.5 mM GcOMe elution, citric acid (CA) elution (remaining antibodies). Image taken at an exposure of 2s.*

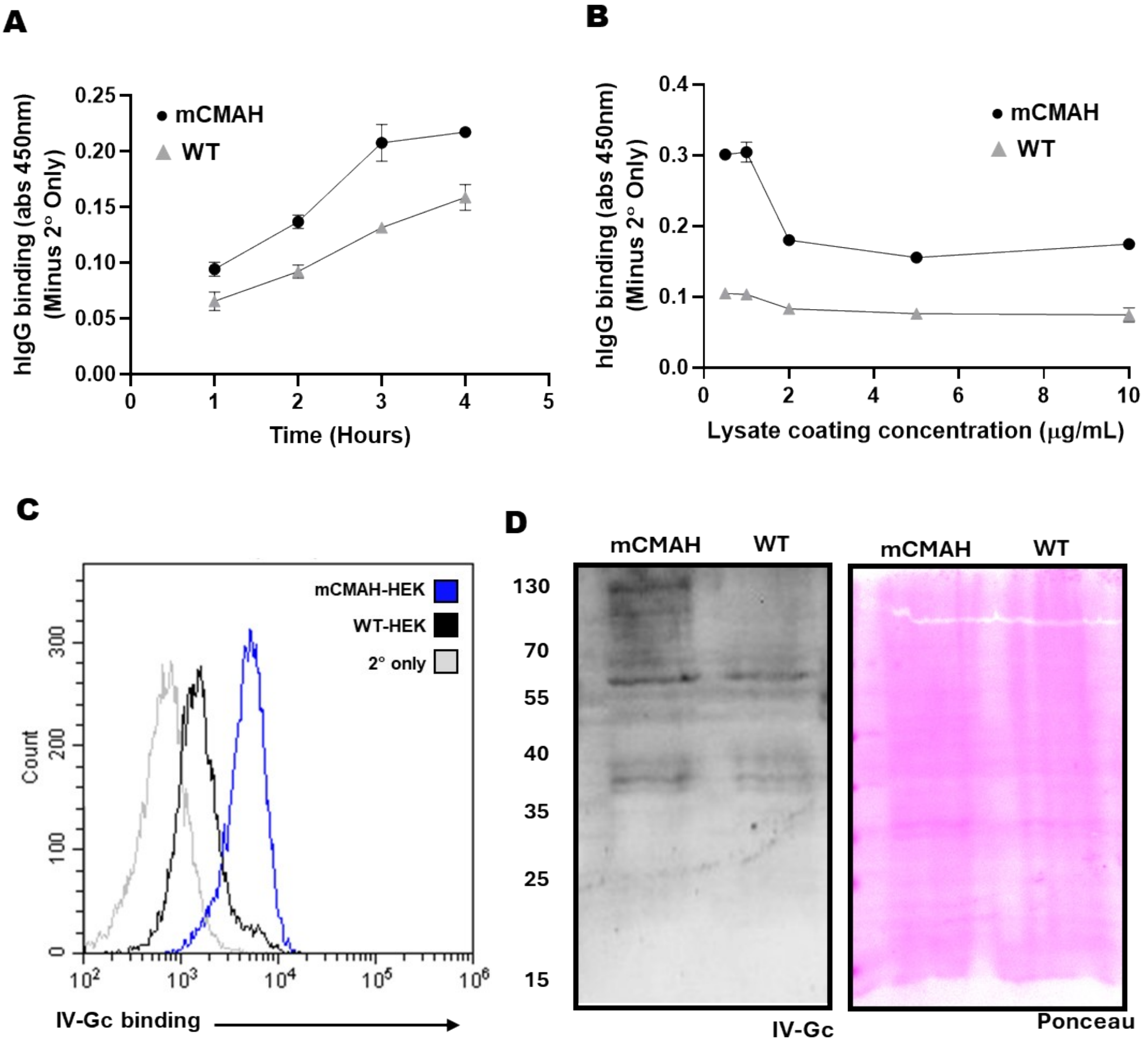

Figure S5: ***Binding of enriched anti-Neu5Gc antibodies from IVIG (IV-Gc) to mCMAH-HEK lysates and cells by ELISA, flow cytometry and western blotting.*** ***(A)*** *Optimisation of IV-Gc binding time for the lysate ELISA. IV-Gc was added at 10 µg/mL and detected using an anti-human IgG antibody. Each point depicts mean ± SD (N=3).* ***(B)*** *Optimisation of lysate coating concentration for IV-Gc binding on the lysate ELISA. Wells of a 96-well ELISA plate were coated with 0.5 to 10 µg/mL mCMAH-HEK or WT-HEK lysates. IV-Gc was added at 10 µg/mL. Each point depicts mean ± SD (N=3).* ***(C)*** *Representative histogram overlay showing binding of IV-Gc to mCMAH-HEK or WT-HEK cells, detected by flow cytometry. IV-Gc was added to cells at 4 µg/mL and detected using a FITC-anti-human IgG/A/M secondary antibody.* ***(D)*** *IV-Gc binding to cell lysates was detected via western blotting. Twenty µg mCMAH-HEK and WT-HEK lysates were run on an SDS-PAGE gel and transferred onto a nitrocellulose membrane. Membranes were stained with ponceau to indicate total lysate. Membranes were then incubated with 10 µg/mL IV-Gc in FG/PBST, followed by HRP-anti-human IgG. Images taken at an exposure time of 15s.*

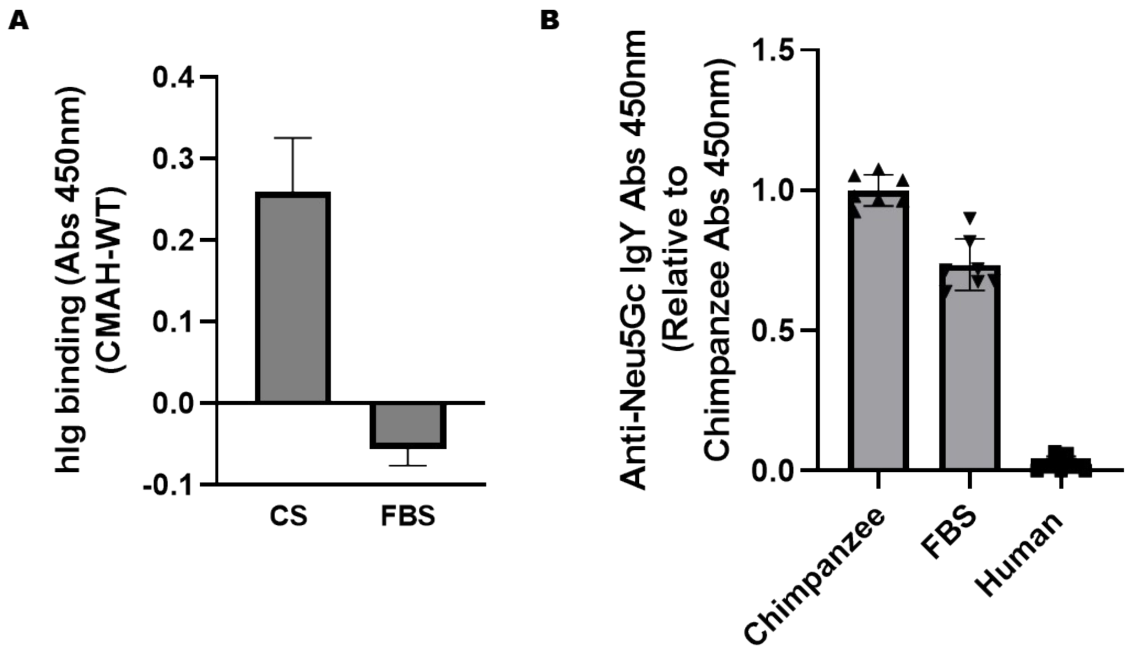

Figure S6: ***Chimpanzee serum cannot be replaced by FBS in the paired column method to enrich anti-Neu5Gc antibodies. (A)*** *Enrichment of anti-Neu5Gc antibodies from IVIG was carried out using resin coupled to chimpanzee serum (CS) or FBS as the solid phase for the Neu5Gc-positive column. IV-Gc was added to the lysate ELISA at 10 µg/mL and detected using an anti-human IgG secondary. Graph depicts mean ± SD (N=3).* ***(B)*** *Neu5Gc is present in chimpanzee serum and FBS, but not in human serum. Serum samples at 1:100 dilution in coating buffer were adsorbed directly onto 96-well ELISA plates. Polyclonal chicken anti-Neu5Gc IgY antibody binding was detected using an anti-chicken HRP secondary antibody. (N=7, 3 independent experiments).*

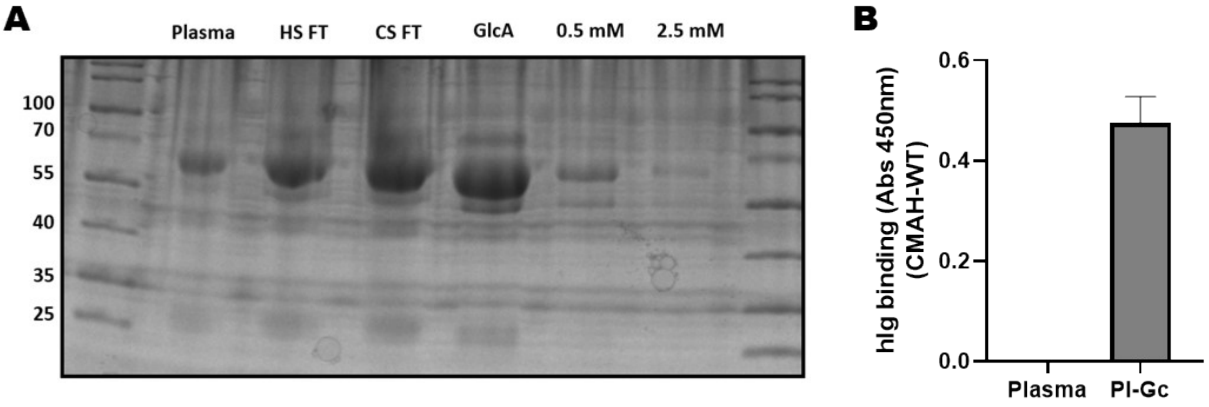

Figure S7: ***Large-scale affinity purification can also be used to isolate anti-Neu5Gc antibodies (PI-Gc) from pooled human plasma. (A)*** *Fractions from the large-scale affinity purification process on pooled plasma from 10 healthy donors were run on an SDS-PAGE gel. Thirty µL of each elution was loaded into each lane. The gel was stained using Coomassie stain and imaged after destaining in 10% acetic acid.* ***From left to right:*** *whole plasma, HS column flowthrough (HS FT), CS column flowthrough (CS FT), Glucuronic acid elution (GlcA), 0.5 mM GcOMe elution, 2.5 mM GcOMe elution.* ***(B)*** *Whole plasma or PI-Gc at a 1:25 dilution were added to the lysate ELISA and binding was detected using an HRP-anti-human IgG secondary. Graph depicts mean ± SD (N=3).*

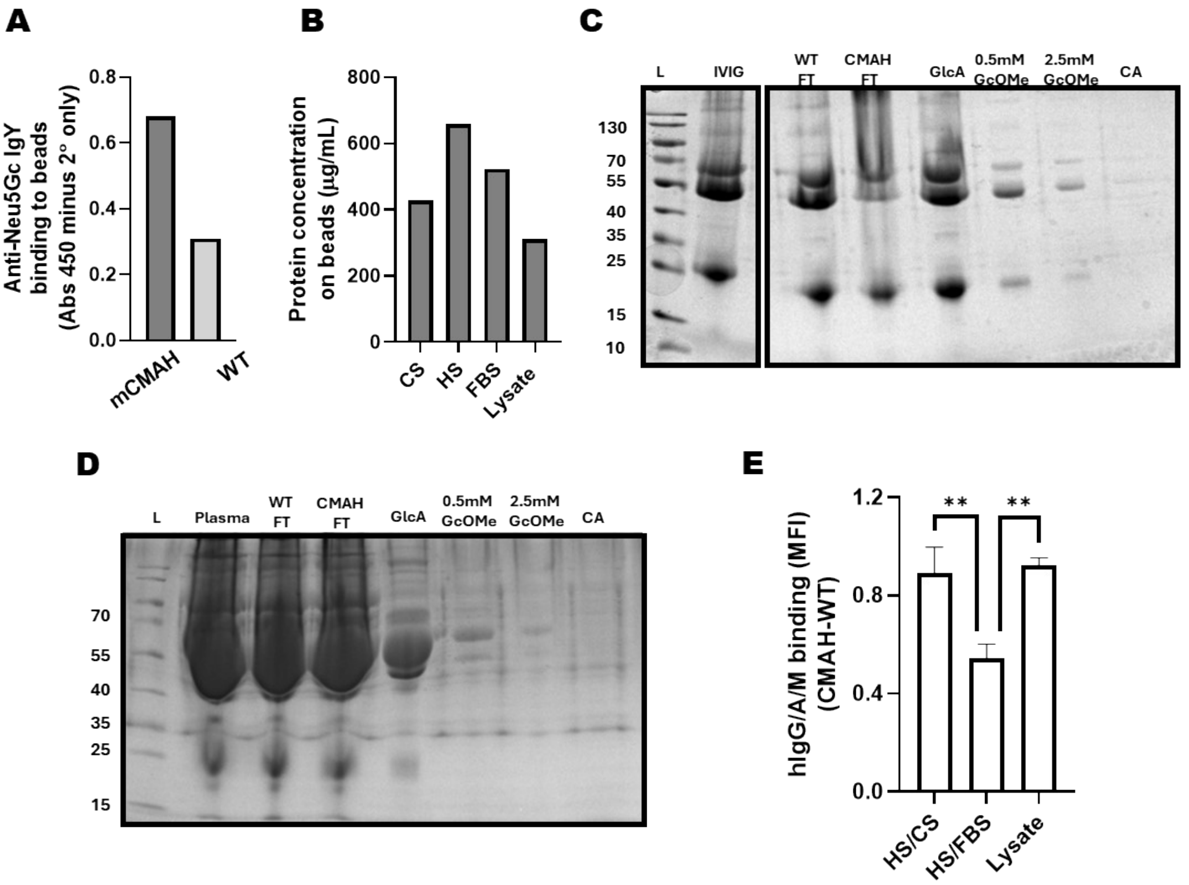

Figure S8: ***Preparation of and enrichment of anti-Neu5Gc antibodies using mCMAH-HEK and WT-HEK lysate columns. (A)*** *Lysate-coupled resin was added to wells of a 96-well plate. Neu5Gc coupled to the resin was detected using anti-Neu5Gc IgY and an HRP-conjugated anti-chicken IgY secondary antibody. (N=2).* ***(B)*** *Protein concentration in resin samples from CS, HS, FBS and lysate-coupled columns was detected via BCA assay. A titration of BSA spiked with resin was used as a standard curve to calculate protein concentration (N=2).* ***(C)*** *Fractions from IVIG enrichment on WT-HEK and mCMAH-HEK columns were run on an SDS-PAGE gel. 30 µL of each fraction was loaded into each lane and visualised using Coomassie stain. From left to right: protein ladder (L), unprocessed IVIG, WT-HEK column flowthrough (WT FT), mCMAH-HEK column flowthrough (CMAH FT), Glucuronic acid elution (GlcA), 0.5 mM GcOMe elution, 2.5 mM GcOMe elution, citric acid elution (CA).* ***(D)*** *Fractions from Pl-Gc from pooled donor plasma enriched using the mCMAH-HEK and WT-HEK lysate columns were run on an SDS-PAGE gel. 30 µL of each fraction was loaded into each lane and visualised using Coomassie stain. From left to right: protein ladder (L), unprocessed plasma, WT-HEK column flowthrough (WT FT), mCMAH-HEK column flowthrough (CMAH FT), Glucuronic acid elution (GlcA), 0.5 mM GcOMe elution, 2.5 mM GcOMe elution, citric acid elution (CA).* ***(E)*** *IV-Gc eluted from the CS/HS and WT/CMAH columns bind to mCMAH-HEK cells via flow cytometry more effectively than when FBS was used as a source of Neu5Gc-containing glycans. Graph depicts Mean ± SD (N=3, one-way ANOVA, P= 0.0012).*
